# Leveraging Natural Compounds for Pancreatic Lipase Inhibition via Virtual Screening

**DOI:** 10.3390/ph18091246

**Published:** 2025-08-22

**Authors:** Emanuele Liborio Citriniti, Roberta Rocca, Claudia Sciacca, Nunzio Cardullo, Vera Muccilli, Francesco Ortuso, Stefano Alcaro

**Affiliations:** 1 Dipartimento di Scienze Della Salute, Università “Magna Græcia” di Catanzaro, Viale Europa, 88100 Catanzaro, Italy; emanueleliborio.citriniti@unicz.it (E.L.C.); ortuso@unicz.it (F.O.); alcaro@unicz.it (S.A.); 2 Net4Science S.r.l., Università “Magna Græcia” di Catanzaro, Viale Europa, 88100 Catanzaro, Italy; 3 Associazione CRISEA—Centro di Ricerca e Servizi Avanzati per l’Innovazione Rurale, Località Condoleo di Belcastro, 88055 Catanzaro, Italy; 4 Dipartimento di Scienze Chimiche, Università Degli Studi di Catania, V.le A. Doria 6, 95125 Catania, Italy; claudia.sciacca@unict.it (C.S.); ncardullo@unict.it (N.C.); vera.muccilli@unict.it (V.M.)

**Keywords:** obesity, pancreatic lipase, docking, molecular dynamics, natural compounds

## Abstract

**Background**: Pancreatic lipase (PL), the principal enzyme catalyzing the hydrolysis of dietary triacylglycerols in the intestinal lumen, is pivotal for efficient lipid absorption and plays a central role in metabolic homeostasis. Enhanced PL activity promotes excessive lipid assimilation and contributes to positive energy balance, key pathophysiological mechanisms underlying the escalating global prevalence of obesity—a complex, multifactorial condition strongly associated with metabolic disorders, including type 2 diabetes mellitus and cardiovascular disease. Inhibition of pancreatic lipase (PL) constitutes a well-established therapeutic approach for attenuating dietary lipid absorption and mitigating obesity. **Methods**: With the aim to identify putative PL inhibitors, a Structure-Based Virtual Screening (SBVS) of PhytoHub database naturally occurring derivatives was performed. A refined library of 10,404 phytochemicals was virtually screened against a crystal structure of pancreatic lipase. Candidates were filtered out based on binding affinity, Lipinski’s Rule of Five, and structural clustering, resulting in six lead compounds. **Results**: In vitro, enzymatic assays confirmed theoretical suggestions, highlighting Pinoresinol as the best PL inhibitor. Molecular dynamics simulations, performed to investigate the stability of protein–ligand complexes, revealed key interactions, such as persistent hydrogen bonding to catalytic residues. **Conclusions**: This integrative computational–experimental workflow highlighted new promising natural PL inhibitors, laying the foundation for future development of safe, plant-derived anti-obesity therapeutics.

## 1. Introduction

Obesity constitutes a multifactorial and escalating global health concern, defined by pathological adipose tissue accumulation that detrimentally impacts metabolic homeostasis and predisposes individuals to a spectrum of comorbidities [1,2]. As a major contributor to lifestyle-associated illnesses such as type 2 diabetes, cardiovascular conditions, and several cancers, it plays a pivotal role in elevating both morbidity and mortality rates across the world [3,4]. The rising global prevalence of obesity underscores the urgent demand for more efficacious therapeutic interventions, as the condition imposes substantial burdens on healthcare infrastructures and is associated with significant impairments in quality of life and overall morbidity. At the core of obesity lies an imbalance in energy homeostasis, where excessive caloric intake, particularly from dietary fat, exceeds the body’s energy expenditure [1,5]. The fat in our diet is primarily composed of mixed triglycerides, which must be cleaved into free fatty acids by lipase enzymes (gastric and pancreatic) before absorption by intestinal enterocytes. Postprandial triglycerides are metabolized by other lipases, such as lipoprotein lipase, hepatic lipase, and endothelial lipase [6]. Among these, pancreatic lipase, secreted by pancreatic acinar cells, is responsible for the complete hydrolysis of dietary fat in the small intestine. This enzyme hydrolyzes triglycerides into monoglycerides and free fatty acids absorbed by the intestinal mucosa. Pancreatic lipase activity is highly efficient, underscoring its critical role in dietary fat metabolism. However, the excessive release of free fatty acids by pancreatic lipase can lead to elevated fat storage in white adipose tissue, contributing to obesity and related metabolic disorders. Its pivotal role in fat metabolism makes it a key contributor to the development of obesity, as excess free fatty acids released into the bloodstream are stored in white adipose tissue, leading to fat accumulation [7]. Consequently, the inhibition of pancreatic lipase (PL) directly targets a root cause of obesity by reducing fat absorption at the level of digestion. This has established PL as a well-recognized therapeutic target for managing obesity and its related metabolic disorders [8].

Currently, orlistat is the only pancreatic lipase (PL) inhibitor approved for clinical use in the therapy of obesity. It exerts its pharmacological effect by inhibiting the enzymatic hydrolysis of dietary triglycerides in the intestinal lumen, thereby reducing fat absorption. Despite its demonstrated efficacy, the application of orlistat is often limited by its adverse gastrointestinal side effects—most notably steatorrhea, abdominal cramping, and bloating—which can substantially impair patient adherence and long-term treatment compliance [9,10,11]. In recent decades, growing scientific interest has been directed toward bioactive natural products as promising alternative inhibitors of PL. Among these, polyphenolic compounds—including flavonoids and isoflavonoids—as well as various terpenoids, have demonstrated notable inhibitory potential, positioning them as promising candidates for safer, plant-derived anti-obesity therapeutics [12,13,14,15]. These phytochemicals inhibit PL activity and provide additional health benefits, such as anti-inflammatory and antioxidant effects. With their structural diversity and bioactivity, natural products offer a promising foundation for developing affordable and safe anti-obesity therapies [16,17].

In silico screening methodologies have significantly advanced drug discovery by enabling high-throughput, structure-based identification of bioactive compounds with enhanced accuracy and efficiency [18,19]. Computational tools, such as molecular docking, molecular dynamics simulations, and pharmacophore modeling, have become indispensable for predicting and elucidating the interactions between natural compounds and molecular targets, such as PL [20,21,22,23]. These approaches substantially expedite early-stage drug discovery by efficiently narrowing down candidate compounds for subsequent in vitro and in vivo validation, reducing both time and resource expenditures. Moreover, virtual screening contributes to the optimization of therapeutic profiles by identifying ligands with enhanced selectivity and reduced off-target liabilities, thereby minimizing the risk of adverse effects [24]. These technologies further support structure-based drug design, facilitating the rational optimization of lead compounds [25,26]. As a result, the application of virtual screening represents a crucial step in developing safe and effective therapies for obesity, harnessing the potential of computational advancements to accelerate the process. In this study, we present a Structure-Based Virtual Screening (SBVS) approach designed to identify novel PL inhibitors from natural compounds as potential anti-obesity agents. By integrating computational techniques with experimental validation, we aim to identify lead compounds derived from nature, laying the groundwork for the development of safe and effective treatments to combat obesity and its associated health risks.

## 2. Results

### 2.1. Computational Identification of Selective Binders

The virtual screening workflow was designed using a rigorous and integrative computational strategy. To evaluate the docking model’s ability to discriminate between active and inactive compounds, key performance metrics were calculated, including Receiver Operating Characteristic (ROC) curve analysis, Area Under the Curve (AUC), and Enrichment Factors (EF) (Figure S1). The docking model demonstrated reliable discriminatory performance, as evidenced by an AUC of 0.75, highlighting its capacity to effectively prioritize bioactive compounds over inactive ones. Predictive accuracy was further validated through EF analysis, which yielded an EF of 5.4 within the top 1% of ranked compounds—corresponding to a retrieval rate exceeding fivefold that expected by random selection. This enrichment performance was maintained across broader selection thresholds, with EFs of 4.9 and 4.8 at the top 2% and 5%, respectively, thereby affirming the model’s robustness and generalizability in virtual screening applications. Further validation of the virtual screening pipeline was achieved through an analysis of the distribution of known active compounds within the ranked list of docked molecules. Remarkably, 24% of the actives were identified within the top 5% of docking scores, and over 50% were located within the top 20%. These findings highlight the pipeline’s ability to effectively enrich for potential inhibitors among high-scoring candidates, supporting its applicability as a reliable tool for early-phase drug discovery efforts targeting PL.

The virtual screening campaign targeted the PhytoHub database [27], which initially comprised 9818 natural product structures. This dataset was expanded to 10,404 unique entries by systematically enumerating all plausible stereoisomers and ionization states at physiological pH (7.4). Orlistat (ORL), a clinically approved inhibitor of PL, was employed as a reference compound to establish a benchmark Glide XP docking score (G-score) of −6.41 Kcal/mol, serving as a threshold for comparative performance evaluation. Compounds scoring better than this threshold were retained for further analyses. Subsequent application of Lipinski’s Rule of Five criteria filtered the dataset down to 603 compounds with favorable drug-like properties [28].

To ensure broad chemical representation within the prioritized hit list, a clustering analysis was performed based on molecular similarity, calculated using Tanimoto coefficients derived from MACCS structural fingerprints. This method allowed the identification of 26 distinct chemical clusters, each representing a unique scaffold and minimizing redundancy within the dataset. For each cluster, the representative compound was defined as the centroid, that is, the molecule with the highest average similarity to all other members of the same cluster. This approach ensured that the selected compounds were structurally representative of their respective chemotypes. Six compounds were ultimately selected for experimental validation based on a combination of G-score, commercial availability, and favorable predicted physicochemical and toxicological profiles. These candidates exhibited strong predicted binding affinities, with G-scores ranging from −7.93 to −10.00 Kcal/mol (Table 1). The remaining compounds were excluded based on predicted cytotoxicity, limited commercial availability, or excessive structural complexity, factors that could compromise assay reliability or increase the likelihood of false-positive results in vitro.

Among the selected hits, Pinoresinol (PHUB001389) from Cluster 16 exhibited the strongest interaction, with a G-score of −10.00 Kcal/mol. Isolariciresinol (PHUB001722) from Cluster 11 also showed a notable binding affinity, with a G-score of −9.49 Kcal/mol. This was closely followed by Dihydroxybergamottin (PHUB000255) from Cluster 20 and Epsilon Viniferin (PHUB000318) from Cluster 14, which had G-scores of −9.23 and −9.14 Kcal/mol, respectively. Archangelicin (PHUB000235) from Cluster 15 displayed a moderate interaction energy of −8.16 Kcal/mol, while Curcumin (PHUB001408) from Cluster 5 recorded a G-score of −7.93 Kcal/mol.

The chemical structure analysis revealed a predominance of polycyclic frameworks, primarily characterized by aromatic systems. Additionally, several identified compounds featured functional groups and heterocyclic motifs such as catechols, furans, benzofurans, and chromenes. These structural elements suggest a significant presence of conjugated π-electron systems and oxygen-containing heterocycles, which could influence the compounds’ chemical reactivity and biological activity.

**Figure 1:**
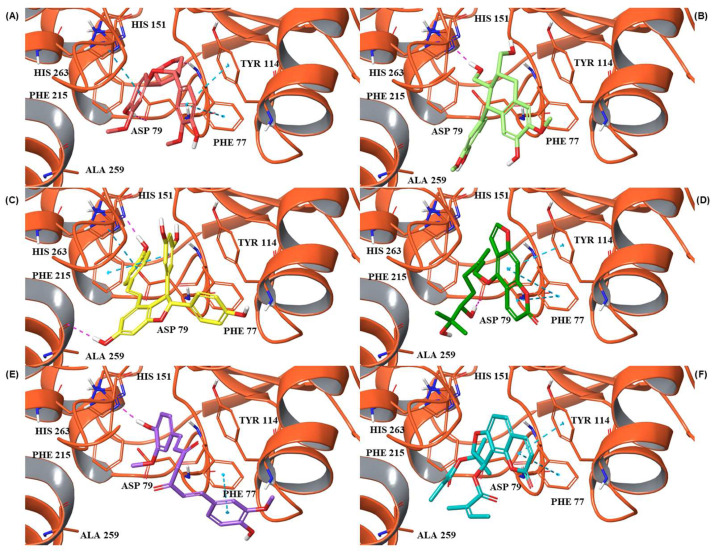
Three-dimensional representations of PL complexed with: (**A**) Pinoresinol (PHUB001389), (**B**) Isolariciresinol (PHUB001722), (**C**) Ɛ-Viniferin (PHUB000318), (**D**) Dihydroxybergamottin (PHUB000255), (**E**) Curcumin (PHUB001408), and (**F**) Archangelicin (PHUB000235). PL is shown as orange cartoons, while ligands are represented as sticks. Amino acid residues involved in key interactions with the ligands are shown as cyan and yellow sticks. Hydrogen bonds and π-π stacking interactions are depicted as yellow and cyan dashed lines, respectively. The figure was created using Maestro (Schrödinger Release 2023-1: Maestro, Schrödinger, LLC., New York, NY, USA, 2023) [29].

For each hit, we analyzed the docking pose of the stereoisomer with the highest G-score for the target. The binding mode analysis revealed that hydrogen bonds (H-bonds) and π-π interactions were the predominant ligand–target interactions across all six hits (Figure 1 and Figure S2).

The binding modes analysis highlighted Pinoresinol (PHUB001389) as the most favorable interacting ligand, achieving a G-score equal to −10.00 Kcal/mol, indicative of strong binding potential and optimal orientation within the active site (Figure 1A and Figure S2A). Its high theoretical affinity can be primarily addressed to two H-bonds: one formed between the ligand furan moiety and the backbone of PHE77, and another between a catechol hydroxyl group and the ASP79 sidechain. Additionally, the catechol rings engage π–π stacking interactions with PHE77, TYR114, and HIS263 sidechains. Notably, both ASP79 and HIS263 are part of the enzyme’s catalytic triad, suggesting that Pinoresinol may directly interfere with the catalytic function of PL. Isolariciresinol (PHUB001722) forms an H-bond with HIS263 through a hydroxyl group on its tetrahydronaphthalenic ring (Figure 1B and Figure S2B). Although π–π stacking interactions are absent in this pose, the specific polar contact likely helps anchor the ligand near the enzymatic core and may contribute to functional inhibition. Ɛ-Viniferin (PHUB000318), a resveratrol oligomer, establishes two key stacking interactions with PHE215 and HIS263 (Figure 1C and Figure S2C). Additionally, its benzofuran moiety forms an H-bond with the backbone of ALA259, providing further stabilization. Dihydroxybergamottin (PHUB000255), a furanocoumarin derivative, interacts with the PL active site through polar and hydrophobic interactions (Figure 1D and Figure S2D). It donates an H-bond to ASP79 via a hydroxyl group on its alkyl side chain and simultaneously forms π–π stacking interactions with PHE77 and TYR114. The proximity of these contacts to functionally relevant residues supports the compound’s potential as a robust enzyme binder. Curcumin (PHUB001408), a well-known polyphenolic compound, exhibits both H-bonding and aromatic stacking (Figure 1E and Figure S2E). A catechol hydroxyl group donates an H-bond to HIS263, while a neighboring aromatic ring engages in π–π stacking with PHE77. Lastly, Archangelicin (PHUB000235) binds primarily via hydrophobic and π–π stacking interactions (Figure 1F and Figure S2F). Its chromene core facilitates two stacking contacts with TYR114 and PHE77. Despite lacking H-bonds, these spatially favorable π–π interactions seem to contribute to ligand stabilization within the active site, suggesting that aromatic stacking alone can sustain effective binding in the absence of polar contacts.

### 2.2. Evaluation of Pancreatic Lipase Inhibitory Activity

The inhibition of PL was evaluated using a previously reported method [20]. The inhibitory activity was expressed as the concentration required to reduce the enzyme activity by 50% (IC_50_ µM), with a lower value indicating greater inhibitory potency. Orlistat, a well-known anti-obesity drug, was used as a positive control. Dose–response curves are reported in Figure S3. Compounds that demonstrated stronger interactions in the SBVS analysis also showed lower IC_50_ values (Table 2). Notably, the lignans (Pinoresinol, Isolariciresinol and **ε**-Viniferin) were the most promising PL inhibitors. Precisely, Pinoresinol and Isolariciresinol (G-score values of −10.00 and −9.49 Kcal/mol, respectively) exhibited the highest inhibitory activity with IC_50_ values of 10.1 and 12.3 µM, followed by ε-viniferin (IC_50_ = 13.2 µM). Whereas molecules, such as Archangelicin (−8.16 Kcal/mol) or Curcumin (−7.93 Kcal/mol), show moderate PL inhibition (IC_50_ = 49.3 and 36.5 µM, respectively).

### 2.3. Molecular Dynamics (MD) Simulations

**Figure 2:**
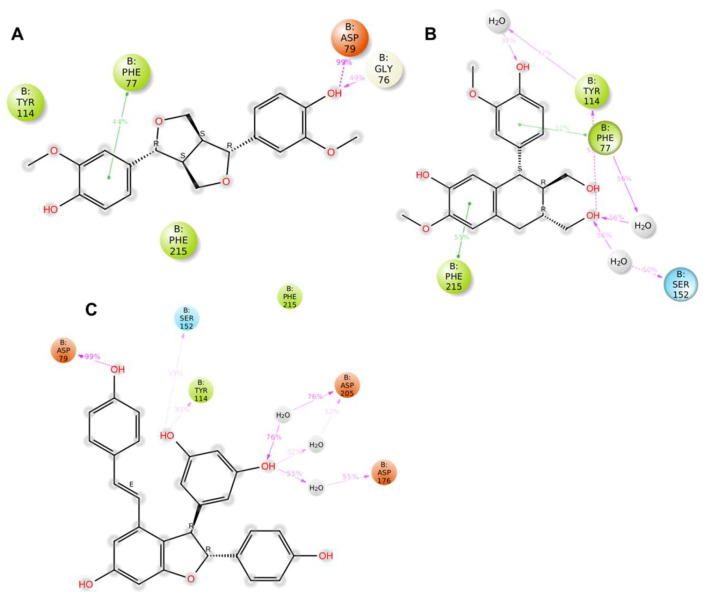
Ligand–residue interaction profiles for pancreatic lipase (PL) in complex with compounds (**A**) Pinoresinol (PHUB001389), (**B**) Isolariciresinol (PHUB001722), and (**C**) ε-Viniferin (PHUB000318) across 200 ns molecular dynamics (MD) simulations. Only interactions that persisted for more than 30% of the 200 ns simulation trajectory are displayed. Hydrogen bonds and π-π stacking interactions are depicted as magenta and green arrows, respectively. The figure was obtained by Maestro (Schrödinger Release 2023-1: Maestro, Schrödinger, LLC., New York, NY, USA, 2023).

Molecular dynamics (MD) simulations were conducted to evaluate the stability of the binding modes of the three most promising hits identified through SBVS and subsequently validated in vitro. These simulations offered critical insights into the dynamic behavior (Figure S4) and interaction profiles of the ligands within the PL active site, highlighting key features relevant to their potential efficacy (Figure 2).

The Root Mean Square Deviation (RMSD) analysis computed during the simulation on the ligands’ heavy atoms reveals an overall higher conformational stability compared to orlistat, which was used as a reference compound (Figure S4). In particular, ε-Viniferin (PHUB000318) (yellow line) displays the most favorable geometric stability profile throughout the 200 ns simulation, maintaining minimal and consistent deviations indicative of a stable and well-oriented binding within the active site. Pinoresinol (PHUB001389) (pink line) and Isolariciresinol (PHUB001722) (green line) exhibit highly similar and overlapping behaviors, with limited fluctuations that further support the structural stability of their respective complexes. These findings suggest that the selected ligands may exhibit stronger and more persistent interactions with the enzyme.

Among the analyzed compounds, Pinoresinol (PHUB001389) exhibited the most stable binding conformation. Key interactions were maintained for over 30% of the MD simulation, with a particularly persistent H-bond between the hydroxyl group of its catechol moiety and the catalytic residue ASP79, found in 99% of the trajectory. Additional stabilizing contacts included a hydrogen bond with GLY76 and a π–π stacking interaction with PHE77, further reinforcing the compound’s affinity and supporting its role as a potent PL inhibitor (Figure 2A).

Isolariciresinol (PHUB001722) also demonstrated a consistent interaction profile, with critical contacts sustained for a significant portion of the simulation. Two prominent water bridges were observed—one with SER152, a member of the catalytic triad, and another with TYR114. The tetrahydronaphthalene moiety of the ligand formed two π–π stacking interactions with PHE215. At the same time, the catechol group established an additional stacking interaction with PHE77, contributing to a stable and well-oriented binding pose.

ε-Viniferin (PHUB000318) displayed a unique and robust interaction pattern. Its phenolic moiety formed a key H-bond with ASP79, while the dihydroxybenzene-furan segment contributed two donor H-bonds with SER152 and TYR114. Moreover, the second hydroxyl group on the dihydroxybenzene ring formed three water bridges with ASP205 and ASP176, enhancing the compound’s overall binding stability.

These MD simulations highlight the strong and specific interactions that underlie the binding stability of the selected compounds. Although in vitro assays indicate moderate yet promising efficacy, the observed interaction patterns suggest that rational structural optimization could further improve their performance. These findings provide a solid foundation for the future development of more potent PL inhibitors with therapeutic potential.

## 3. Discussion

In this study, molecular modeling techniques were employed to identify natural compounds with potential inhibitory activity against PL, a key therapeutic target in obesity management. Out of an extensive library of bioactive phytochemicals, six top-ranking hits were selected on the basis of their docking scores and interaction profiles. Among them, three compounds—Pinoresinol (PHUB001389), Isolariciresinol (PHUB001722), and ε-Viniferin (PHUB000318)—emerged as the most promising candidates, demonstrating significant PL inhibition in vitro with IC_50_ values in the low micromolar range.

Notably, these three molecules are naturally occurring polyphenols widely distributed in Mediterranean dietary sources, which adds translational relevance to our findings [30,31,32]. Pinoresinol, a lignan primarily found in extra virgin olive oil, has previously been reported to possess antioxidant, anti-inflammatory, and cardioprotective properties [33]. Our computational and experimental data further support its bioactivity, revealing a strong and stable binding affinity to the catalytic triad of PL. This dual evidence of enzymatic inhibition and dietary prevalence reinforces its potential as a nutraceutical component for obesity prevention.

Similarly, Isolariciresinol, another lignan found abundantly in whole grains, legumes, and seeds such as flaxseed, is metabolized by gut microbiota into enterolignans with phytoestrogenic activity [34,35,36]. Although its interaction profile with PL was less extensive than that of Pinoresinol, it retained key hydrogen bonding with catalytically relevant residues, suggesting it could still serve as a valuable scaffold for further optimization.

ε-Viniferin, a resveratrol oligomer highly concentrated in red wine, grapes, and grape-derived products, displayed the most stable binding profile during molecular dynamics simulations. Its robust π–π stacking and multiple hydrogen bonding interactions suggest a strong and persistent occupancy of the PL active site. These results are in agreement with the literature data reporting its anti-inflammatory, antioxidant, and metabolic regulatory properties, extending its known bioactivity spectrum to include PL inhibition [37].

From a nutritional standpoint, it is particularly noteworthy that all three lead compounds are naturally enriched in the typical foods of the Mediterranean diet—a dietary pattern long associated with reduced obesity and metabolic disease risk [38,39,40]. Their presence in staple components such as olive oil, whole grains, and red wine highlights a potential synergistic mechanism by which adherence to the Mediterranean diet may confer anti-obesity benefits [41,42,43].

While the general anti-obesity potential of Ɛ-viniferin has been explored in previous studies [44], its specific role as pancreatic lipase (PL) inhibitors remains relatively under-investigated. Some reports mention related derivatives, such as glycosylated forms of pinoresinol, in the context of plant extracts with lipase-inhibitory effects. However, the direct activity of the free compounds—particularly pinoresinol and ε-viniferin—has not been thoroughly characterized [45].

Other studies have examined the broader metabolic or anti-adipogenic effects of ε-viniferin, often employing assay formats different from those used in the current work and lacking detailed mechanistic insights into PL binding. Additionally, some reviews focus on unrelated polyphenols, such as curcuminoids, without addressing pinoresinol or ε-viniferin at all [14].

In this context, our study provides complementary and novel data by employing a combined computational and experimental strategy to assess the PL inhibitory potential of these two natural compounds. Molecular docking and dynamics simulations, together with enzymatic assays, revealed that both pinoresinol and ε-viniferin form stable and specific interactions within the active site of PL, correlating with measurable in vitro inhibition.

Given their natural origin, documented bioactivity, and low micromolar potency, these compounds represent attractive candidates for development as dietary supplements aimed at obesity prevention or adjunctive therapy. Furthermore, their well-defined chemical scaffolds and favorable pharmacological profiles provide a solid foundation for medicinal chemistry efforts to optimize their potency, selectivity, and bioavailability. Structural modifications aimed at enhancing lipase-binding affinity or metabolic stability could generate novel lead compounds for anti-obesity drug development.

In conclusion, the integration of molecular modeling and experimental evaluation has successfully identified food-derived polyphenols with PL inhibitory activity. These findings support further exploration of Pinoresinol, Isolariciresinol, and ε-Viniferin as functional food components or nutraceuticals and also as valuable leads for pharmacological optimization in the context of obesity management.

## 4. Materials and Methods

### 4.1. Database Preparation

The PhytoHub database (https://phytohub.eu, accessed on 1 January 2024) [27], comprising 9818 phytochemicals, was retrieved as the initial compounds’ virtual library. Ligands preparation was conducted using LigPrep [46] at physiological pH (7.4), incorporating protonation state prediction, tautomeric enumeration, and stereoisomer. This preprocessing step yielded a dataset of 10,404 distinct molecular entities, which were subsequently employed in molecular docking simulations to evaluate their binding potential against the selected target.

### 4.2. Receptor Preparation

The crystallographic structure of the PL (PDB ID: 1LPB), a model extensively employed in structure-based drug design [18,47,48], was retrieved from the Protein Data Bank (PDB) [49]. This 2.46 Å resolution crystal structure, which represents human pancreatic lipase in complex with porcine colipase, was prepared for docking studies by removing the colipase molecule, as it does not interact with the lipase active site [50]. This structure comprises two co-crystallized ligands: methoxy undecyl phosphonic acid (MUP), an alkyl phosphonate mechanism-based inhibitor covalently bound to the catalytic serine residue (SER152), and β-octylglucoside (BOG), a surfactant molecule used to stabilize the enzyme. To enable unbiased docking simulations, both ligands were removed during the receptor preparation phase.

The protein structure was refined using the Protein Preparation Wizard in Maestro [47], which assigned protonation states appropriate for physiological pH (7.4), corrected bond orders, and added missing hydrogen atoms. Missing side chains and loop regions were modeled to ensure structural integrity. Finally, a restrained energy minimization was performed using the OPLS_2005 force field to relieve steric clashes and optimize the overall geometry of the protein [51]. Following these preprocessing steps, the G-score of the reference inhibitor orlistat was calculated to establish a benchmark affinity threshold for evaluating ligand–target interactions in subsequent molecular docking experiments.

### 4.3. Structure-Based Virtual Screening (SBVS)

In this study, we performed a Structure-Based Virtual Screening (SBVS), using the Glide software (Glide, Schrödinger Release 2020-4) [52]. The receptor grid was constructed by centering it at the geometric centroid of the key catalytic residues ASP79, SER152, and HIS263, with the covalently bound co-crystallized inhibitor excluded from the model to avoid bias in docking simulations. This choice was based on site-directed mutagenesis studies of the cDNA encoding human pancreatic lipase, which identified SER152, HIS263, and ASP176 as the key residues forming the catalytic triad required for enzymatic activity. The triad follows a serine protease-like catalytic mechanism. Functional assays have demonstrated that the substitution of either SER152 or HIS263 results in a complete loss of catalytic activity, confirming their indispensable roles in the hydrolytic mechanism. Conversely, mutation of ASP176 to glutamate retains approximately 80% of enzymatic activity, indicating a degree of structural and functional tolerance at this position within the triad [53]. The inner box dimensions of the receptor grid were defined as 15 × 15 × 15 Å to ensure thorough spatial coverage of the active site cavity. It has been specifically designed to target the active site where orlistat interacts, allowing for precise simulation of molecular interactions. Molecular docking simulations were performed using the Glide at extra precision (XP) mode [54], employing the OPLS2005 force field. Under these conditions, orlistat exhibited a predicted binding affinity of −6.41 Kcal/mol. To evaluate the robustness and discriminative power of the docking protocol, a validation study was conducted using a dataset comprising 225 experimentally confirmed actives from BindingDB [55] (Table S1) and 4914 decoys generated from DUD-E (Directory of Useful Decoys, Enhanced) [56]. The performance of the virtual screening workflow was assessed using standard statistical metrics, including the Receiver Operating Characteristic (ROC) curve, Area Under the Curve (AUC), and Enrichment Factor (EF).

### 4.4. Post-Docking Filtering and Clustering for Hit Selection

After docking, the resulting ligands were filtered according to Lipinski’s Rule to retain compounds exhibiting favorable drug-like properties. This filtering step ensured compliance with key pharmacokinetic parameters, including acceptable molecular weight (≤500 Da), lipophilicity (LogP ≤ 5), hydrogen bond donors (≤5), hydrogen bond acceptors (≤10), and, where applicable, a suitable topological polar surface area (TPSA), consistent with oral bioavailability criteria [28].

Subsequently, the filtered compounds were clustered based on structural similarity using MACCS molecular fingerprints and the Tanimoto similarity coefficient. This chemoinformatic approach enabled the identification of distinct structural scaffolds, thereby facilitating the prioritization of representative compounds for downstream analysis and potential experimental validation. Each cluster was subsequently analyzed via visual inspection to ensure scaffold diversity and chemical tractability. From these, six representative compounds—defined as the centroids of their respective chemical clusters—were selected for further consideration based on a combination of high docking scores (G-score), favorable physicochemical and toxicological profiles, and confirmed commercial availability.

### 4.5. In Vitro Pancreatic Lipase Inhibition

The inhibition studies of porcine pancreatic lipase (triacylglycerol acyl hydrolase; EC 3.1.1.3) were performed employing 4-nitrophenyl butyrate as a substrate [20]. The compounds were dissolved in MeOH at concentrations from 0.22 mM to 2.7 mM. Orlistat, employed as a positive reference, was prepared at 6.7 μM in phosphate buffer (PBS). The assay was performed in a 96-well microplate by adding PBS (150 μL of 50 mM, pH = 7.2), PL solution (15 μL of 5 mg/mL in phosphate buffer), and different aliquots (2–10 μL) of tested compounds or orlistat and incubating at 37 °C for 10 min. Then, the substrate solution was added (10 μL of 3.2 mM in H_2_O:DMF, 70:30), and the plate was incubated and shaken for 30 min at 37 °C. The optical density values (*OD*) were acquired at 405 nm with a Synergy H1 microplate reader (BioTek, Bad Friedrichshall, Germany). The experiments were performed in triplicate, and the %inhibition was determined by the following equation:

(1) $$\%inhibition=\frac{OD_{control}-{OD}_{sample}}{{OD}_{control}}\times \;100\%$$

where *OD_control_* refers to the enzyme–substrate mixture, while *OD_sample_* corresponds to the enzyme–substrate mixture in the presence of the inhibitor. Inhibition data were evaluated as a dose–response curve to determine the IC_50_, the concentration of inhibitor that reduces enzyme activity by 50%.

OriginPro 2023 software (OriginLab Corp., Northampton, MA, USA) was adopted for statistical analysis. The regression coefficients were determined based on the analysis of variance (one-way ANOVA, significance level 0.01). A *p*-value < 0.01 was set as the threshold for significance for Tukey’s test.

### 4.6. Molecular Dynamics Simulations (MD)

The complexes of the target protein with the best-performing compounds, as determined by enzymatic assays, were subjected to 200 ns of molecular dynamics (MD) simulations using Desmond [57]. Each molecular system was enclosed in an orthorhombic simulation box with a 10 Å buffer and solvated using the TIP3P water model (Transferable Intermolecular Potential with 3 Points) [58]. To neutralize the system’s net charge, five Na^+^ counterions were introduced. OPLS 2005 force field was applied for energy estimation. The solvated systems underwent energy minimization to remove steric clashes and ensure optimal starting configurations, followed by relaxation using the Martyna–Tobias–Klein isobaric–isothermal (MTK_NPT) ensemble. Equilibration was performed in two phases: first, under an NVT ensemble (canonical ensemble) at 10 K to stabilize initial configurations, and then under an NPT ensemble (isothermal–isobaric ensemble) at 300 K and 1 atm pressure, employing the Berendsen thermostat and barostat to maintain temperature and pressure conditions. System trajectories were recorded at 200 ps intervals and analyzed using the Simulation Interaction Diagram and Simulation Event Analysis tools. These analyses offered comprehensive insights into the structural properties of the protein–ligand complexes, highlighting critical aspects such as key binding interactions, Root Mean Square Deviation (RMSD), Root Mean Square Fluctuation (RMSF), and the temporal stability of hydrogen bonding patterns. By capturing these dynamic interaction profiles, this integrative approach provided a robust and systematic evaluation of the binding stability and interaction dynamics of the lead compounds.

## 5. Conclusions

Through an integrated computational and experimental approach, three natural polyphenols—Pinoresinol, Isolariciresinol, and ε-Viniferin—were identified as promising PL inhibitors with low micromolar potency and stable binding profiles. These compounds, derived from Mediterranean dietary sources, exhibited strong in vitro inhibitory activity and favorable interaction with catalytically relevant PL residues, underscoring their therapeutic potential. Their dual role as both functional food components and pharmacologically active molecules highlights a translational opportunity for obesity management. While promising, further studies are warranted to assess their in vivo efficacy, pharmacokinetics, and potential for structural optimization. Overall, these findings position the three polyphenols as compelling leads for the development of nutraceuticals or novel anti-obesity therapeutics.

## Figures and Tables

**Table 1:** Cluster number, PHUB ID, common name, 2D structure, and G-score values (Kcal/mol) for the top six compounds proposed as potential pancreatic lipase (PL) inhibitors.

Cluster Number	PhytoHub ID Database	Common Name	2D Structure	G-Score (Kcal/mol)
**5**	PHUB001408	Curcumin	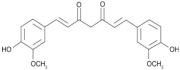	−7.93
**15**	PHUB000235	Archangelicin	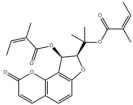	−8.16
**14**	PHUB000318	Ɛ-Viniferin	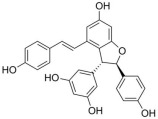	−9.13
**20**	PHUB000255	Diidroxybergamottin	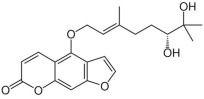	−9.23
**11**	PHUB001722	Isolariciresinol	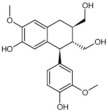	−9.49
**16**	PHUB001389	Pinoresinol	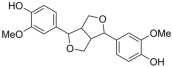	−10.00

**Table 2:** IC_50_ values of selected molecules inhibiting pancreatic lipase (PL). The IC_50_ values are means ± SD (*n* = 3). Compounds are listed in order of increasing IC_50_ values. The most interesting compounds are shown in italics, and orlistat (positive control) in bold.

Compound	IC_50_ (µM)
**Orlistat**	0.15 ± 0.02 ^e^
*Pinoresinol*	10.1 ± 0.3 ^d^
*Isolariciresinol*	12.3 ± 1.2 ^d^
**ε** *-* *Viniferin*	13.2 ± 0.6 ^d^
Archangelicin	49.3 ± 1.4 ^b^
Curcumin	36.5 ± 4.8 ^c^
Diidroxybergamottin	84.5 ± 1.5 ^a^

^a,b,c,d,e^ The data were statistically analyzed by One-way ANOVA with a significance level set at 0.01. Values with the same letters are not significantly different at *p* < 0.01 (Tukey test).

## Data Availability

The data are contained within the article or Supplementary Materials.

## Supplementary Materials

The following supporting information can be downloaded at https://www.mdpi.com/article/10.3390/ph18091246/s1: Table S1: Active compounds targeting pancreatic lipase. BindingDB codes and canonical SMILES of active compounds against pancreatic lipase (PL), used as positive controls in enrichment factor studies; Figure S1: ROC curve comparing Glide XP enrichment performance to a random model. Receiver Operating Characteristic (ROC) curve for the enrichment study obtained using the Glide XP protocol (blue line) compared to a random model (gray line). The ROC curve demonstrates the trade-off between sensitivity (true positive rate) and specificity (false positive rate); Figure S2: Two-dimensional interaction diagrams of pancreatic lipase with selected natural inhibitors. Two-dimensional representations of PL complexed with (A) Pinoresinol (PHUB001389), (B) Isolariciresinol (PHUB001722), (C) Ɛ-Viniferin (PHUB000318), (D) Dihydroxybergamottin (PHUB000255), (E) Curcumin (PHUB001408), and (F) Archangelicin (PHUB000235). Hydrogen bonds and π-π stacking interactions are depicted as magenta and green lines, respectively; Figure S3: Dose–response curves of orlistat and selected compounds for pancreatic lipase inhibition. Dose–response curves for the pancreatic lipase inhibitory activity of orlistat and selected compounds. Data points are obtained according to Equation (1) reported in the manuscript; Figure S4: RMSD trend of selected ligands in complex with pancreatic lipase during MD simulations. Root mean square deviation (RMSD) profiles of Orlistat (blue line), Pinoresinol (PHUB001389) (pink line), Isolariciresinol (PHUB001722) (green line), and Ɛ-Viniferin (PHUB000318) (yellow line) in complex with pancreatic lipase (PL). RMSD values were calculated for the ligand heavy atoms over the course of molecular dynamic (MD) simulations, following superposition of the protein backbone.

## Abbreviations

The following abbreviations are used in this manuscript:

PL	Pancreatic Lipase
SBVS	Structure-Based Virtual Screening
ROC	Receiver Operating Characteristic
AUC	Area Under the Curve
EF	Enrichment Factors
ORL	Orlistat
G-Score	Glide XP Docking Score
IC_50_	Half Maximal Inhibitory Concentration
MD	Molecular Dynamics
RMSD	Root Mean Square Deviation
PDB	Protein Data Bank
OPLS	Optimized Potentials for Liquid Simulations
DUD-E	Directory of Useful Decoys, Enhanced
TPSA	Topological Polar Surface Area
PBS	Phosphate Buffer
OD	Optical Density
MTK_NPT	Martyna–Tobias–Klein for NPT Ensemble
NVT	Canonical Ensemble
NPT	Isothermal–Isobaric Ensemble
RMSF	Root Mean Square Fluctuation

## References

[1] Lingvay I., Cohen R.V., Roux C.W.L., Sumithran P. (2024). Obesity in adults. Lancet.

[2] Gilden A.H., Catenacci V.A., Taormina J.M. (2024). Obesity. Ann. Intern. Med..

[3] Welsh A., Hammad M., Piña I.L., Kulinski J. (2024). Obesity and cardiovascular health. Eur. J. Prev. Cardiol..

[4] Gonzalez-Gutierrez L., Motiño O., Barriuso D., de la Puente-Aldea J., Alvarez-Frutos L., Kroemer G., Palacios-Ramirez R., Senovilla L. (2024). Obesity-Associated Colorectal Cancer. Int. J. Mol. Sci..

[5] Wang H., He W., Yang G., Zhu L., Liu X. (2024). The Impact of Weight Cycling on Health and Obesity. Metabolites.

[6] Omer E., Chiodi C. (2024). Fat digestion and absorption: Normal physiology and pathophysiology of malabsorption, including diagnostic testing. Nutr. Clin. Pract..

[7] Ren C., Cao Z., Liu Y., Wang R., Lin C., Wang Z. (2024). Medicinal chemistry aspects of fat mass and obesity associated protein: Structure, function and inhibitors. Future Med. Chem..

[8] Kumar A., Chauhan S. (2021). Pancreatic lipase inhibitors: The road voyaged and successes. Life Sci..

[9] Feng X., Lin Y., Zhuo S., Dong Z., Shao C., Ye J., Zhong B. (2023). Treatment of obesity and metabolic-associated fatty liver disease with a diet or orlistat: A randomized controlled trial. Am. J. Clin. Nutr..

[10] Heck A.M., Yanovski J.A., Calis K.A. (2000). Orlistat, a new lipase inhibitor for the management of obesity. Pharmacotherapy.

[11] Filippatos T.D., Derdemezis C.S., Gazi I.F., Nakou E.S., Mikhailidis D.P., Elisaf M.S. (2008). Orlistat-associated adverse effects and drug interactions: A critical review. Drug Saf..

[12] Bialecka-Florjanczyk E., Fabiszewska A.U., Krzyczkowska J., Kurylowicz A. (2018). Synthetic and Natural Lipase Inhibitors. Mini Rev. Med. Chem..

[13] Faraone I., Russo D., Genovese S., Milella L., Monné M., Epifano F., Fiorito S. (2021). Screening of in vitro and in silico α-amylase, α-glucosidase, and lipase inhibitory activity of oxyprenylated natural compounds and semisynthetic derivatives. Phytochemistry.

[14] He X.Q., Zou H.D., Liu Y., Chen X.J., Atanasov A.G., Wang X.L., Xia Y., Ng S.B., Matin M., Wu D.T. (2024). Discovery of Curcuminoids as Pancreatic Lipase Inhibitors from Medicine-and-Food Homology Plants. Nutrients.

[15] Chen Z.Q., He W.Y., Yang S.Y., Ma H.H., Zhou J., Li H., Zhu Y.D., Qian X.K., Zou L.W. (2024). Discovery of natural anthraquinones as potent inhibitors against pancreatic lipase: Structure-activity relationships and inhibitory mechanism. J. Enzyme Inhib. Med. Chem..

[16] Buchholz T., Melzig M.F. (2015). Polyphenolic Compounds as Pancreatic Lipase Inhibitors. Planta Med..

[17] Cardullo N., Muccilli V., Pulvirenti L., Tringali C. (2021). Natural Isoflavones and Semisynthetic Derivatives as Pancreatic Lipase Inhibitors. J. Nat. Prod..

[18] Maruca A., Ambrosio F.A., Lupia A., Romeo I., Rocca R., Moraca F., Talarico C., Bagetta D., Catalano R., Costa G. (2020). 12. Computer-based techniques for lead identification and optimization I: Basics. Fundamental Concepts.

[19] Lupia A., Moraca F., Bagetta D., Maruca A., Ambrosio F.A., Rocca R., Catalano R., Romeo I., Talarico C., Ortuso F. (2020). Computer-based techniques for lead identification and optimization II: Advanced search methods. Phys. Sci. Rev..

[20] Sciacca C., Cardullo N., Pulvirenti L., Di Francesco A., Muccilli V. (2023). Evaluation of honokiol, magnolol and of a library of new nitrogenated neolignans as pancreatic lipase inhibitors. Bioorg. Chem..

[21] Cardullo N., Calcagno D., Pulvirenti L., Sciacca C., Pittalà M.G.G., Maccarronello A.E., Thevenard F., Muccilli V. (2024). Flavonoids with lipase inhibitory activity from lemon squeezing waste: Isolation, multispectroscopic and in silico studies. J. Sci. Food Agric..

[22] Yang L., Cao S., Xie M., Shi T. (2024). Virtual screening, activity evaluation, and stability of pancreatic lipase inhibitors in the gastrointestinal degradation of nattokinase. Heliyon.

[23] Yuan Y., Pan F., Zhu Z., Yang Z., Wang O., Li Q., Zhao L. (2023). Construction of a QSAR Model Based on Flavonoids and Screening of Natural Pancreatic Lipase Inhibitors. Nutrients.

[24] Zloh M., Kirton S.B. (2018). The benefits of in silico modeling to identify possible small-molecule drugs and their off-target interactions. Future Med. Chem..

[25] Schierle S., Neumann S., Heitel P., Willems S., Kaiser A., Pollinger J., Merk D. (2020). Design and Structural Optimization of Dual FXR/PPARδ Activators. J. Med. Chem..

[26] Dong L., Shen S., Jiang X., Liu Y., Li J., Chen W., Wang Y., Shi J., Liu J., Ma S. (2022). Discovery of Azo-Aminopyrimidines as Novel and Potent Chitinase O. J. Agric. Food Chem..

[27] da Silva A.B., Giacomoni F., Pavot B., Fillatre Y., Rothwell J., Sualdea B.B., Veyrat C., Garcia-Villalba R., Gladine C., Kopec R. PhytoHub V1.4: A new release for the online database dedicated to food phytochemicals and their human metabolites. Proceedings of the 1. International Conference on Food Bioactives & Health.

[28] Lipinski C.A., Lombardo F., Dominy B.W., Feeney P.J. (2001). Experimental and computational approaches to estimate solubility and permeability in drug discovery and development settings. Adv. Drug. Deliv. Rev..

[29] (2023). Maestro.

[30] Visioli F., Galli C. (2001). The role of antioxidants in the Mediterranean diet. Lipids.

[31] Scalbert A., Manach C., Morand C., Rémésy C., Jiménez L. (2005). Dietary polyphenols and the prevention of diseases. Crit. Rev. Food Sci. Nutr..

[32] Pérez-Jiménez J., Neveu V., Vos F., Scalbert A. (2010). Identification of the 100 richest dietary sources of polyphenols: An application of the Phenol-Explorer database. Eur. J. Clin. Nutr..

[33] Romani A., Ieri F., Urciuoli S., Noce A., Marrone G., Nediani C., Bernini R. (2019). Health Effects of Phenolic Compounds Found in Extra-Virgin Olive Oil, By-Products, and Leaf of *Olea europaea* L.. Nutrients.

[34] Adlercreutz H. (2007). Lignans and human health. Crit. Rev. Clin. Lab. Sci..

[35] De Silva S.F., Alcorn J. (2019). Flaxseed Lignans as Important Dietary Polyphenols for Cancer Prevention and Treatment: Chemistry, Pharmacokinetics, and Molecular Targets. Pharmaceuticals.

[36] Rowland I., Faughnan M., Hoey L., Wähälä K., Williamson G., Cassidy A. (2003). Bioavailability of phyto-oestrogens. Br. J. Nutr..

[37] Xue Y.Q., Di J.M., Luo Y., Cheng K.J., Wei X., Shi Z. (2014). Resveratrol oligomers for the prevention and treatment of cancers. Oxid. Med. Cell Longev..

[38] Buckland G., Bach A., Serra-Majem L. (2008). Obesity and the Mediterranean diet: A systematic review of observational and intervention studies. Obes. Rev..

[39] Estruch R., Ros E., Salas-Salvadó J., Covas M.I., Corella D., Arós F., Gómez-Gracia E., Ruiz-Gutiérrez V., Fiol M., Lapetra J. (2013). Retraction and Republication: Primary Prevention of Cardiovascular Disease with a Mediterranean Diet. N. Eng. J. Med..

[40] Tosti V., Bertozzi B., Fontana L. (2018). Health Benefits of the Mediterranean Diet: Metabolic and Molecular Mechanisms. J. Gerontol. Ser. A.

[41] Martínez-González M.A., Gea A., Ruiz-Canela M. (2019). The Mediterranean Diet and Cardiovascular Health. Circ. Res..

[42] Kastorini C.M., Milionis H.J., Esposito K., Giugliano D., Goudevenos J.A., Panagiotakos D.B. (2011). The effect of Mediterranean diet on metabolic syndrome and its components: A meta-analysis of 50 studies and 534,906 individuals. J. Am. Coll. Cardiol..

[43] Bach-Faig A., Berry E.M., Lairon D., Reguant J., Trichopoulou A., Dernini S., Medina F.X., Battino M., Belahsen R., Miranda G. (2011). Mediterranean diet pyramid today. Science and cultural updates. Public Health Nutr..

[44] Ohara K., Kusano K., Kitao S., Yanai T., Takata R., Kanauchi O. (2015). ε-Viniferin, a resveratrol dimer, prevents diet-induced obesity in mice. Biochem. Biophys. Res. Commun..

[45] Ji Y.L., Feng X., Chang Y.Q., Zheng Y.G., Hou F.J., Zhang D., Guo L. (2024). Chemical characterization of different parts of Forsythia suspensa and α-glucosidase and pancreatic lipase inhibitors screening based on UPLC-QTOF-MS/MS and plant metabolomics analysis. Arab. J. Chem..

[46] Schrödinger, LLC (2018). LigPrep.

[47] Yadav M., Singh V.P. (2023). Glutathione Peroxidase-like Antioxidant Activity of 1,3-Benzoselenazoles: Synthesis and In Silico Molecular Docking Studies as Pancreatic Lipase Inhibitors. J. Org. Chem..

[48] Samira N., Khedidja B., Zahra A.F., Elyakine C.K.N., Mohamed Y. (2020). In silico and in vitro Study of the Inhibitory Effect of Antiinflammatory Drug Betamethasone on Two Lipases. Antiinflamm. Antiallergy Agents Med. Chem..

[49] Berman H.M., Westbrook J., Feng Z., Gilliland G., Bhat T.N., Weissig H., Shindyalov I.N., Bourne P.E. (2000). The Protein Data Bank. Nucleic Acids Res..

[50] van Tilbeurgh H., Egloff M.P., Martinez C., Rugani N., Verger R., Cambillau C. (1993). Interfacial activation of the lipase–procolipase complex by mixed micelles revealed by X-ray crystallography. Biochemistry.

[51] (2018). Protein Preparation Wizard.

[52] Shivakumar D., Harder E., Damm W., Friesner R.A., Sherman W. (2012). Improving the Prediction of Absolute Solvation Free Energies Using the Next Generation OPLS Force Field. J. Chem. Theory Comput..

[53] Lowe M.E. (1997). Structure and function of pancreatic lipase and colipase. Annu. Rev. Nutr..

[54] (2018). Glide.

[55] Liu T., Hwang L., Burley S.K., Nitsche C.I., Southan C., Walters W.P., Gilson M.K. (2025). BindingDB in 2024: A FAIR knowledgebase of protein-small molecule binding data. Nucleic Acids Res..

[56] Huang N., Shoichet B.K., Irwin J.J. (2006). Benchmarking sets for molecular docking. J. Med. Chem..

[57] Schrödinger (2021). Desmond Molecular Dynamics System ver. 4.4.

[58] Nayar D., Agarwal M., Chakravarty C. (2011). Comparison of Tetrahedral Order, Liquid State Anomalies, and Hydration Behavior of mTIP3P and TIP4P Water Models. J. Chem. Theory Comput..

