# Supplementary material for: Leveraging Natural Compounds for Pancreatic Lipase Inhibition via Virtual Screening
Source: Pharmaceuticals (Basel). 2025 Aug 22;18(9):1246. doi: 10.3390/ph18091246 (PMC12472586; doi:10.3390/ph18091246)
Supplement: Supplementary file 1 [file pharmaceuticals-18-01246-s001.zip › pharmaceuticals-3792410-supplementary.pdf]

Supplementary Material for

Leveraging Natural Compounds for Pancreatic Lipase Inhibition via Virtual Screening

Emanuele Liborio Citriniti [1], Roberta Rocca [1,2,3,*], Claudia Sciacca [4], Nunzio Cardullo [4], Vera Muccilli [4], Francesco Ortuso [1,2] and Stefano Alcaro [1,2,3]

1 Dipartimento di Scienze Della Salute, Università "Magna Græcia" di Catanzaro, Viale Europa, 88100 Catanzaro, Italy; emanueleliborio.citriniti@unicz.it (E.L.C.); ortuso@unicz.it (F.O.); alcaro@unicz.it (S.A.)
2 Net4Science S.r.l., Università "Magna Græcia" di Catanzaro, Viale Europa, 88100 Catanzaro, Italy
3 Associazione CRISEA—Centro di Ricerca e Servizi Avanzati per l'Innovazione Rurale, Località Condoleo di Belcastro, 88055 Catanzaro, Italy
4 Dipartimento di Scienze Chimiche, Università Degli Studi di Catania, V.le A. Doria 6, 95125 Catania, Italy; claudia.sciacca@unict.it (C.S.); ncardullo@unict.it (N.C.); vera.muccilli@unict.it (V.M.)
* Correspondence: rocca@unicz.it

Table S1. **Active Compounds Targeting Pancreatic Lipase.** BindingDB codes and canonical SMILES of active compounds against pancreatic lipase (PL), used as positive controls in enrichment factor studies

Figure S1. ROC Curve Comparing Glide XP Enrichment Performance to a Random Model. Receiver Operating Characteristic (ROC) curve for the enrichment study obtained using the Glide XP protocol (blue line) compared to a random model (gray line). The ROC curve demonstrates the trade-off between sensitivity (true positive rate) and Specificity (false positive rate).

Academic Editors: George Mihai Nitulescu, Dragos Mihai and Robert Vianello

Received: 16 July 2025
Revised: 7 August 2025
Accepted: 11 August 2025

Published: 22 August 2025

Citation: Citriniti, E.L.; Rocca, R.; Sciacca, C.; Cardullo, N.; Muccilli, V.; Ortuso, F.; Alcaro, S. Leveraging Natural Compounds for Pancreatic Lipase Inhibition via Virtual Screening. *Pharmaceuticals* **2025**, *18*, 1246. https://doi.org/10.3390/ph18091246

Copyright: © 2025 by the authors. Licensee MDPI, Basel, Switzerland. This article is an open access article distributed under the terms and conditions of the Creative Commons Attribution (CC BY) license (https://creativecommons.org/licenses/by/4.0/).

Figure S2: 2D Interaction Diagrams of Pancreatic Lipase with Selected Natural Inhibitors. 2D representations of PL complexed with **A)** Pinoresinol (PHUB001389), **B)** Isolariciresinol (PHUB001722), **C)** Ɛ-Viniferin (PHUB000318), **D)** Dihydroxybergamottin (PHUB000255), **E)** Curcumin (PHUB001408), and **F)** Archangelicin (PHUB000235). Hydrogen bonds and π-π stacking interactions are depicted as magenta and green lines, respectively.

Figure S3: Dose–Response Curves of Orlistat and Selected Compounds for Pancreatic Lipase Inhibition. Dose-response curves for the pancreatic lipase inhibitory activity of orlistat and selected compounds. Data points are obtained according to equation (1) reported in the manuscript.

Figure S4. RMSD Trend of Selected Ligands in Complex with Pancreatic Lipase During MD Simulations. Root-mean-square deviation (RMSD) profiles of Orlistat (blue line), Pinoresinol (PHUB001389) (pink line), Isolariciresinol (PHUB001722) (green line), and Ɛ-Viniferin (PHUB000318) (yellow line) in complex with pancreatic lipase (PL). RMSD values were calculated for the ligand heavy atoms over the course of molecular dynamics (MD) simulations, following superposition of the protein backbone.

Table S1. **Active Compounds Targeting Pancreatic Lipase.** BindingDB codes and canonical SMILES of active compounds against pancreatic lipase (PL), used as positive controls in enrichment factor studies

**BindingDB**	**SMILES chemical formula**
Morin	Oc1cc(O)cc(c12)oc(c(c2=O)O)-c3c(O)cc(O)cc3
BindingDB_50401727_3D	Oc1cc(O)cc(c12)occc2=O
Gen	Oc1cc(O)cc(c12)occ(c2=O)-c3ccc(O)cc3
BindingDB_50401723_3D	CC(C)=CCc(c1O)c(O)cc(c12)occ(c2=O)-c3ccc(O)cc3
BindingDB_50401724_3D	c1cc(O)c(OC)cc1-c(c2=O)coc(c23)cc(O)cc3O
Biochanin	COc(cc1)ccc1-c(c2=O)coc(c23)cc(O)cc3O
BindingDB_51366121_3D	c1cccc(F)c1/C=C(/C2=O)Cc(c23)ccc(c3)O
BindingDB_51366118_3D	c1cccc(Cl)c1/C=C(/C2=O)Cc(c23)ccc(c3)O
BindingDB_51366113_3D	c1cc(O)ccc1/C=C(/C2=O)Cc(c23)ccc(c3)O
Naringenin	Oc1cc(O)cc(c12)O[C@@H](CC2=O)c3ccc(O)cc3
BindingDB_50401719_3D	Oc1c(O)c(O)cc(c12)O[C@@H]([C@H](C2=O)O)c3ccc(O)cc3
BindingDB_50381000_mol_1	COc(cc1)c(O)cc1-c(c(c2=O)O)oc(c23)cc(O)cc3
BindingDB_50380999_mol_1	COc(cc1)ccc1-c(c(c2=O)O)oc(c23)cc(O)cc3
BindingDB_50380998_mol_1	COc1c(O)cc(cc1)[C@@H](CC2)Oc(c23)cc(O)cc3
BindingDB_50240348_mol_1	Oc1cc(O)cc(c12)O[C@@H]([C@H](C2=O)O)c3ccc(O)cc3
BindingDB_51282461_3D	Oc1c(O)ccc(c1)[C@H]([C@H](C2)O)Oc(c23)c(c(O)cc3O)Cc(c4O)c(O)cc(c45)O[C@@H]([C@H](C5)O)c(c6)ccc(O)c6O
BindingDB_51282464_3D	Oc1c(O)ccc(c1)[C@H]([C@H](C2)O)Oc(c23)c(c(O)cc3O)Cc(c(O)cc4O)c(c45)O[C@@H]([C@H](C5)O)c(c6)ccc(O)c6O
BindingDB_50213686_m	[C@@H]12[C@@H]3[C@@H](O3)[C@@](O)(C(=O)[C@@H]1O2)C[C@@H](C(=O)OC)NC(=O)/C=C/CCCC/C=C/C

ol_1	
BindingDB_50542118_3D	C1=CC(C)(C)Oc(c(C)c2)c1c3[nH]c(c4c23)ccc(c4)OC
BindingDB_50346601_mol_1	CC(C)(C1)CC[C@](C(=O)O)(CC2)[C@@H]1C([C@@]23C)=CC[C@H]4[C@@]3(C)CC[C@@H]5[C@]4(C)CC[C@H](O)C5(C)C
BindingDB_50337360_mol_1	O=C1C[C@@H]([C@@H](O1)C)c2cc(OC)c(O)cc2
BindingDB_50392475_mol_1	c1cc(O)c(O)cc1\C=C\C(=O)O[C@@H](C(=O)O)[C@@H](O)COC(=O)c2cc(O)c(O)c(c2)O
BindingDB_50067040_mol_1	c1cc(O)c(OC)cc1\C=C\C(=O)CC(=O)/C=C/c2cc(OC)c(O)cc2
BindingDB_50337364_mol_1	O=C(O)c1cc(OC)c(O)cc1
BindingDB_50851656_3D	c1cc(O)ccc1CCC(=O)c2c(O)c(c(O)cc2O[C@H](O3)[C@H](O)[C@@H](O)[C@H](O)[C@H]3CO)Cc(c(O)c4C(=O)CCc5ccc(O)cc5)c(O)cc4O[C@H](O6)[C@H](O)[C@@H](O)[C@H](O)[C@H]6CO
BindingDB_50213694_mol_1	[C@H]12[C@H]3[C@H](O3)[C@@](O)(C(=O)[C@H]1O2)C[C@H](C(=O)OC)NC(=O)/C=C/CCCC/C=C/C
BindingDB_50392474_mol_1	O[C@@H]1CO[C@H]([C@H](O)[C@H]1O)O[C@H]([C@@H](O)[C@@H](O)[C@H]2CO)[C@@H](O2)Oc(c3O)c(-c4cc(O)c(O)cc4)oc(c35)cc(O)cc5=O
1	c1cc(O)ccc1CCC(=O)c2c(cc(O)cc2O)O[C@H](O3)[C@H](O)[C@@H](O)[C@H](O)[C@H]3CO
**2**	Oc1cc(O)cc(c12)O[C@@H]([C@H](C2)O)c(c3)ccc(O)c3O
BindingDB_50542117_3D	C1=CC(C)(C)Oc(c(C)c2)c1c3[nH]c(c4c23)cc(OC)c(c4)OC
BindingDB_50135527_mol_1	C=C(C)[C@@H](C1)Oc(c12)ccc3c2O[C@H]4[C@@H](C3=O)c5c(OC4)cc(OC)c(c5)OC
BindingDB_4375_mol_1	O=C(O)/C=C/c1cc(O)c(O)cc1
BindingDB_50392477_mol_1	O=C(O)[C@H](O)[C@@H](O)CO
BindingDB_50085536_mol_1	Oc1cc(C(=O)O)cc(O)c1O
BindingDB_51277070_3D	NS(=O)(=O)NCc(o1)nnc1[C@H](S(=O)(=O)C)c(n2)sc(c23)cc(cc3)-c4ccccc4
BindingDB_51366122_3D	c1cc(O)c(OC)cc1/C=C(/C2=O)Cc(c23)ccc(c3)OC
BindingDB_50542104_3D	COc1c(OC)ccc(c1)NC(=O)C(=O)c(c2)ccc3[nH]c(c4c23)cccc4
BindingDB_51282462_3D	Oc1c(O)ccc(c1)[C@H]([C@H](C2)O)Oc(c23)c(c(O)cc3O)Cc(c4O)c(O)c(c(c45)O[C@@H]([C@H](C5)O)c(c6)ccc(O)c6O)Cc(c(O)cc7O)c(c78)O[C@@H]([C@H](C8)O)c(c9)ccc(O)c9O
BindingDB_	c1ccccc1N(C)C(=O)n(n2)c(SCC(F)(F)F)nc2-c3ccc(Cl)cc3

50138737_mol_1	
BindingDB_50138751_mol_1	c1ccccc1N(C)C(=O)n(n2)c(SC)nc2-c3ccc(cc3)OC(F)(F)F
BindingDB_50138735_mol_1	CC(C)c1c(C)n(oc1=O)C(=O)N(C2)CCC[C@@H]2C
BindingDB_429816_3D	c1cc(O)c(OC)cc1CC[C@@H](O)C[C@H](O)CCc2cc(OC)c(O)cc2
BindingDB_429815_3D	c1cc(O)ccc1CC[C@@H](O)C[C@H](O)CCc2ccc(O)cc2
BindingDB_50259746_mol_1	c1cc(O)c(OC)cc1\C=C\C(=O)OCCc2ccc(O)cc2
Untitled	c1c(O)cc(OC)c2OCCc(c3c12)cccc3
Untitled	c1cccc(c1c23)CCOc2c(O)cc(c3)O
BindingDB_429805_3D	COc1cc(OC)cc2ccc(c3c12)cc(c(c3)O)OC
BindingDB_429801_3D	c1ccccc1CCc2cc(OC)cc(c2)OC
BindingDB_429802_3D	c1cccc(O)c1CCc2cc(OC)c(c(c2)O)OC
Phloretin	Oc1cc(O)cc(O)c1C(=O)CCc2ccc(O)cc2
BindingDB_50401712_3D	c1cc(O)ccc1[C@H](CC2=O)Oc(c23)c(CC=C(C)C)c(O)c(c3O)Cc4ccc(O)cc4
BindingDB_50542107_3D	CN(C)c1ccc(cc1)NC(=O)C(=O)c(c2)ccc3n(CC)c(c4c23)cccc4
BindingDB_50333303_3D	c1cc(O)cc(c12)O[C@@H](CC2=O)c3c(O)cc(O)cc3
BindingDB_50333306_3D	c1cc(O)c(O)c(c12)oc(c2)-c3cc(O)cc(c3)O
BindingDB_50333310_3D	c1c(O)c(O)cc(c12)oc(c2)-c3cc(O)cc(c3)O
BindingDB_50442403_mol_1	CC(C)=CCc(c1O)c(O)c(CC=C(C)C)c(c12)occ(c2=O)-c3ccc(O)cc3
BindingDB_50337361_mol_1	O=C1C[C@H]([C@@H](O1)C)c2cc(OC)cc(c2)O
BindingDB_50241243_mol_1	OC[C@@H]1[C@@H](O)[C@H](O)[C@@H](O)[C@@H](O1)Oc(c2=O)c(-c3ccc(O)cc3)oc(c24)cc(O)cc4O
BindingDB_50241354_mol_1	OC[C@@H]1[C@@H](O)[C@H](O)[C@@H](O)[C@@H](O1)Oc(c2=O)c(-c3cc(O)c(O)cc3)oc(c24)cc(O)cc4O
BindingDB_50333297_3D	OC[C@@H]1[C@@H](O)[C@H](O)[C@@H](O)[C@@H](O1)Oc(c2=O)c(-c3ccc(cc3)OC)oc(c24)cc(O)cc4O
BindingDB_50269605_mol_1	Oc1cc(O)cc(c12)O[C@H](CC2=O)c3c(O)cc(O)cc3

BindingDB_50303002_mol_1	CC(C)=CCC/C(C)=C/Cc1c(cc(O)cc1O)-c(c2)oc(c23)cc(O)cc3
BindingDB_50333319_3D	o1ccc(c2)c1cc(c23)oc(c3)-c4cc(O)cc(c4)O
BindingDB_50269559_mol_1	Oc1cc(O)cc(c12)oc(cc2=O)-c3c(O)cc(O)cc3
Isorhamnetin	c1cc(O)c(OC)cc1-c(c(c2=O)O)oc(c23)cc(O)cc3O
kaempferol	Oc1cc(O)cc(c12)oc(c(c2=O)O)-c3ccc(O)cc3
quercetin	Oc1cc(O)cc(c12)oc(c(c2=O)O)-c3cc(O)c(O)cc3
BindingDB_50108046_mol_1	c1cc(O)cc(O)c1/C=C/c2cc(O)cc(c2)O
BindingDB_50250915_mol_1	c1cc(O)cc(c12)oc(c2)-c3cc(O)cc(c3)O
BindingDB_50177405_mol_1	O=Cc1cc(OC)c(O)cc1
BindingDB_51366119_3D	c1cc(Cl)c(Cl)cc1/C=C(/C2=O)Cc(c23)ccc(c3)O
BindingDB_50380997_mol_1	c1cc(O)ccc1CCCc2c(OC)cc(O)cc2
BindingDB_51366117_3D	c1cc(Br)ccc1/C=C(/C2=O)Cc(c23)ccc(c3)O
BindingDB_50401716_3D	CC(C)=CCc(c(O)cc1O)c(c12)O[C@H](CC2=O)c3ccc(O)cc3
BindingDB_51282460_3D	Oc1c(O)ccc(c1)[C@H]([C@H](C2)O)Oc(c23)cc(O)c(c3O)Cc(c4O)c(O)cc(c45)O[C@@H]([C@H](C5)O)c(c6)ccc(O)c6O
BindingDB_50401717_3D	Oc1cc(O)cc(c12)O[C@@H](CC2=O)c3cc(O)cc(c3)O
BindingDB_51366133_3D	C=CCCCCCCCCNc(c1)ccc(c12)CC(/C2=O)=C\c3cc(OC)c(O)cc3
5j	c1ccccc1C(=O)/C=C/c2ccccc2
BindingDB_50401713_3D	CC(C)(O)CCc(c(O)cc1O)c(c12)O[C@H](CC2=O)c(c3)c(O)cc(c34)OC(C)(C)C=C4
BindingDB_50241625_mol_1	c1cc(O)cc(c12)O[C@@H](CC2)c3ccc(O)cc3
BindingDB_50251012_mol_1	c1cc(O)ccc1CCCc2c(O)cc(cc2)OC
BindingDB_50401728_3D	CC(O1)(C)C=Cc(c2O)c1cc(c23)occc3=O
BindingDB_	Cc1ccc(cc1)NC(=O)C(=O)c(c2)ccc3n(c(c4c23)cccc4)Cc5ccc(Cl)cc5

50542102_3D	
BindingDB_50542103_3D	c1ccccc1NC(=O)C(=O)c(c2)ccc3n(c(c4c23)cccc4)Cc5ccc(Cl)cc5
BindingDB_51098598_3D	O=CCC(\C=O)=C/C[C@@H]1C(=C)CC[C@H]([C@@]12C)C(C)(C)CCC2
BindingDB_51134808_3D	CC(C)=CCCC(=C)[C@@H](O)Cc(c(O)c1C(=O)C(C)C)c(O)c(c1O)CC(=C2O)C(=O)C(=C(O)C2(C)C)C(=O)[C@H](C)CC
BindingDB_50851655_3D	c1cc(O)ccc1CCC(=O)c2c(O)c(c(O)cc2O[C@H](O3)[C@H](O)[C@@H](O)[C@H](O)[C@H]3CO)Cc(c(O)cc4O)c(O)c4C(=O)CCc5ccc(O)cc5
BindingDB_50542113_3D	CN(C)c1ccc(cc1)NC(=O)C(=O)c(c2)ccc3n(c(c4c23)cccc4)Cc5ccc(Cl)cc5
BindingDB_51366111_3D	c1c(O)ccc(c12)CC(/C2=O)=C\c3cc(OC)c(OC)c(c3)OC
BindingDB_51366116_3D	c1ccc(Br)cc1/C=C(/C2=O)Cc(c23)ccc(c3)O
BindingDB_50542124_3D	COc(cc1)ccc1NC(=O)C(=O)c(c2)ccc3n(c(c4c23)cccc4)Cc5ccc(Cl)cc5
BindingDB_51366107_3D	c1cc(O)ccc1/C=C(/C2=O)Cc(c23)cccc3
BindingDB_50890321_3D	O=C(S1)NC(=O)\C1=C\c2c(-c(cc3)ccc3C)nn(c2)-c4ccccc4
BindingDB_50542106_3D	c1ccnc(c12)ccc(c2)NC(=O)C(=O)c(c3)ccc4n(CC)c(c5c34)cccc5
BindingDB_50326047_mol_1	O=C(O)[C@@H](CS)NC(=O)[C@@H]([C@@H](C)O)NC(=O)[C@@H](CCC(=O)N)NC(=O)CNC(=O)[C@H]1CCCN1C(=O)[C@H](Cc2cnc[nH]2)NC(=O)[C@@H]3CCCN3C(=O)[C@H](CCC(=O)N)NC(=O)[C@@H](N)CS
BindingDB_50542115_3D	COc1c(OC)ccc(c1)NC(=O)C(=O)c(c2)ccc3n(CC)c(c4c23)cccc4
BindingDB_50890313_3D	O=C(S1)NC(=O)\C1=C\c2c(-c3ccc([N+]([O-])=O)cc3)nn(c2)-c4ccccc4
BindingDB_50542119_3D	CC(C)=CCC[C@](C)(C=C1)Oc(c(C)c2)c1c3[nH]c(c4c23)cccc4
BindingDB_50242015_mol_1	c1cc(O)cc(O)c1-c(c(c2=O)CC=C(C)C)oc(c23)c(CC=C(C)C)c(O)cc3O
BindingDB_51282463_3D	Oc1c(O)ccc(c1)[C@H]([C@H](C2)O)Oc(c23)c(c(O)cc3O)Cc(c4O)c(O)c(c(c45)O[C@@H]([C@H](C5)O)c(c6)ccc(O)c6O)Cc(c(c78)O[C@@H]([C@H](C8)O)c(c9)ccc(O)c9O)c(O)c(c7O)Cc(c(O)cc1O)c(c12)O[C@@H]([C@H](C2)O)c(c1)ccc(O)c1O
BindingDB_50890324_3D	O=C(S1)NC(=O)\C1=C\c2c(-c3ccccc3)nn(c2)-c4ccccc4
BindingDB_	COc(cc1)ccc1NC(=O)C(=O)c(c2)ccc3[nH]c(c4c23)cccc4

50542110_3D	
BindingDB_50542129_3D	c1cnccc1NC(=O)C(=O)c(c2)ccc3n(c(c4c23)cccc4)Cc5ccc(Cl)cc5
BindingDB_51366112_3D	c1c(O)ccc(c12)CC(/C2=O)=C\c3c(OC)cc(OC)cc3OC
BindingDB_50542112_3D	c1ccnc(c12)ccc(c2)NC(=O)C(=O)c(c3)ccc4n(c(c5c34)cccc5)Cc6ccc(Cl)cc6
BindingDB_50542123_3D	COc1c(OC)cc(cc1OC)NC(=O)C(=O)c(c2)ccc3n(C)c(c4c23)cccc4
BindingDB_50542120_3D	c1ccccc1NC(=O)C(=O)c(c2)ccc3n(CC)c(c4c23)cccc4
BindingDB_51366110_3D	COc(cc1)cc(OC)c1/C=C(/C2=O)Cc(c23)ccc(c3)O
BindingDB_51366129_3D	C=CCCCCNc(c1)ccc(c12)CC(/C2=O)=C\c3cc(OC)c(O)cc3
BindingDB_50392476_mol_1	c1cc(O)c(O)cc1\C=C\C(=O)O[C@H]([C@@H](O)C(=O)O)COC(=O)c2cc(O)c(O)c(c2)O
BindingDB_50542116_3D	c1ccc(OC)cc1NC(=O)C(=O)c(c2)ccc3n(c(c4c23)cccc4)Cc5ccc(Cl)cc5
BindingDB_50213689_mol_1	[C@@H]12[C@@H]3[C@@H](O3)[C@](O)(C(=O)[C@@H]1O2)C[C@@H](C(=O)OC)NC(=O)/C=C/CCCC/C=C/C
BindingDB_50542114_3D	CC(C)=CCC/C(C)=C/CCC(C(=O)O1)=CC12c3c(ccc(c3)O)OC(=O)C2(CC(=O)c4c(O)ccc(c4)O)CC\C=C(C)\CCC=C(C)C
BindingDB_50429042_mol_1	c1cc(F)ccc1NC(=O)C(=O)c(c2)ccc3n(c(c4c23)cccc4)Cc5ccc(Cl)cc5
BindingDB_392707_3D	O[C@H](O1)[C@@](O2)(O)[C@H]2C[C@@]13c4c(c5c(cc4)O[C@H](C5)C(=C)C)O[C@H]6[C@@H]3c7c(OC6)cc(OC)c(c7)OC
BindingDB_50381001_mol_1	O[C@@H](O1)[C@](O2)(O)[C@@H]2C[C@@]13c4c(c5c(cc4)O[C@H](C5)C(=C)C)O[C@H]6[C@@H]3c7c(OC6)cc(OC)c(c7)OC
BindingDB_51366115_3D	C1=CC(=O)C=C(OC)[C@]1(O)CCCc2ccc(O)cc2
BindingDB_50333324_3D	c1cccc(Br)c1/C=C(/C2=O)Cc(c23)ccc(c3)O
BindingDB_50542108_3D	CC(O1)(C)C=Cc(c2)c1cc(c23)oc(c3)-c4cc(O)cc(c4)O
BindingDB_50251014_mol_1	c1cnccc1NC(=O)C(=O)c(c2)ccc3[nH]c(c4c23)cccc4
BindingDB_50542125_3	CC(C)=CCc(c1)c(O)cc(c12)oc(c2)-c3cc(O)cc(c3)O

D	
BindingDB_50381284_mol_1	c1ccccc1NC(=O)C(=O)c(c2)ccc3[nH]c(c4c23)cccc4
BindingDB_51366120_3D	CC(C)=CCc1c(O)cc(cc1O)-c(c2)oc(c23)cc(O)cc3
BindingDB_50542111_3D	c1cc(F)ccc1/C=C(/C2=O)Cc(c23)ccc(c3)O
BindingDB_50542128_3D	Cc1cnc(s1)NC(=O)C(=O)c(c2)ccc3n(c(c4c23)cccc4)Cc5ccc(Cl)cc5
BindingDB_51366114_3D	c1ccc(OC)cc1NC(=O)C(=O)c(c2)ccc3[nH]c(c4c23)cccc4
BindingDB_50371232_mol_1	c1c(O)ccc(c12)CC(/C2=O)=C\c3c(OC)cc(OC)c(c3)OC
BindingDB_50542127_3D	Oc1c(O)c(C(C)C)cc(c1[C@]23C(=O)O)CC[C@H]2C(C)(C)CCC3
BindingDB_50686041_3D	c1ccccc1NC(=O)C(=O)c(c2)ccc3n(C)c(c4c23)cccc4
	CC(C)=CC[C@@]12[C@@H](OC1=O)CC(=C2)C(OC)OC
BindingDB_50576045_3D	c1cc(O)c(OC)cc1/C=C(/C2=O)Cc(c23)ccc(c3)N4CCCCC4
BindingDB_51366126_3D	CCCCCC[C@H](C1=C)[C@@H](O1)C[C@@H](OC(=O)[C@@H](NC=O)CC(C)C)CCCCCCCCCCC
BindingDB_51409224_3D	c1cc(O)c(OC)cc1/C=C(/C2=O)Cc(c23)ccc(c3)N4CCCC4
BindingDB_51098590_3D	C#CCOc(cc1)ccc1C(=O)O[C@H](C[C@H](O2)[C@@H](C2=O)CCCCCC)CCCCCCCCCCC
BindingDB_51409225_3D	C1CCC(C)(C)[C@@H]([C@]12C)CCC(=C)[C@H]2C\C=C(\C=O)CC(=O)OCc3cn(nn3)Cc4c(Br)ccc(c4)OC
BindingDB_50890318_3D	CCCCCC[C@H](C1=O)[C@@H](O1)C[C@@H](OC(=O)c2cc(OC)ccc2)CCCCCCCCCCC
BindingDB_50851652_3D	c1ccccc1-n(c2)nc(-c3ccc(Cl)cc3)c2/C=C4/C(=O)N(C(=O)S4)Cc5ccc([N+]([O-])=O)cc5
BindingDB_51098585_3D	c1cc(O)ccc1CCC(=O)c2c(O)c(c(O)cc2O)Cc(c(O)cc3O)c(O)c3C(=O)CCc4ccc(O)cc4
BindingDB_50890317_3D	C1CCC(C)(C)[C@@H]([C@]12C)CCC(=C)[C@H]2C\C=C(\C=O)CC(=O)OCc3cn(nn3)Cc4ccccc4
BindingDB_51409227_3D	c1ccccc1-n(c2)nc(-c3ccc(F)cc3)c2/C=C4/C(=O)N(C(=O)S4)Cc5ccc([N+]([O-])=O)cc5
BindingDB_	CCCCCC[C@H](C1=O)[C@@H](O1)C[C@@H](OC(=O)c2ccc(cc2)OC)CCCCCCCCCCC

50251013_mol_1	
BindingDB_51098580_3D	CC(C)=CCc(c(O)cc1)c(O)c1C(=O)/C=C/c2c(O)cc(O)cc2
BindingDB_392706_3D	C1CCC(C)(C)[C@@H]([C@]12C)CCC(=C)[C@H]2C\C=C(\C=O)CC(=O)OCc3cn(nn3)CC(=O)c4ccccc4
BindingDB_50542131_3D	C=C(C)[C@@H](C1)Oc(cc2)c1c(c2[C@]34CO4)O[C@H]5[C@@H]3c6c(OC5)cc(OC)c(c6)OC
BindingDB_50193719_mol_1	COc1c(OC)ccc(c1)NC(=O)C(=O)c(c2)ccc3n(c(c4c23)cccc4)Cc5ccc(Cl)cc5
BindingDB_51366106_3D	CC(C)=CCc(c1O)c(O)cc(c12)O[C@@H](CC2=O)c(c3)c(O)cc(c34)OC(C)(C)C=C4
	c1cc(O)c(OC)cc1/C=C(/C2=O)Cc(c23)cccc3
BindingDB_51134807_3D	C#CCOC(=O)CC(\C=O)=C/C[C@@H]1C(=C)CC[C@H]([C@@]12C)C(C)(C)CCC2
BindingDB_50890319_3D	CC(C)=CCC/C(C)=C/Cc(c(O)c1C(=O)C(C)C)c(O)c(c1O)CC(=C2O)C(=O)C(=C(O)C2(C)C)C(=O)[C@H](C)CC
BindingDB_51366124_3D	c1ccccc1-n(c2)nc(-c3ccc([N+]([O-])=O)cc3)c2/C=C4/C(=O)N(C(=O)S4)Cc5ccc([N+]([O-])=O)cc5
BindingDB_50542132_3D	c1cc(O)c(OC)cc1/C=C(/C2=O)Cc(c23)ccc(c3)OC(C)C
BindingDB_50193723_mol_1	COc1c(OC)cc(cc1OC)NC(=O)C(=O)c(c2)ccc3n(c(c4c23)cccc4)Cc5ccc(Cl)cc5
BindingDB_50686039_3D	CC(C)=CCc(c1O)c(O)cc(c12)O[C@@H](CC2=O)c(c3)c(O)cc(O)c3CC=C(C)C
BindingDB_50337363_mol_1	OC/C(C)=C/C[C@@]12[C@@H](OC1=O)CC(=C2)CO
BindingDB_50890316_3D	C[C@H](O)[C@@H](O)c1cc(OC)c(O)cc1
BindingDB_50542109_3D	c1ccccc1-n(c2)nc(-c3ccc(cc3)OC)c2/C=C4/C(=O)N(C(=O)S4)Cc5ccc([N+]([O-])=O)cc5
BindingDB_50890315_3D	COc1c(OC)cc(cc1OC)NC(=O)C(=O)c(c2)ccc3[nH]c(c4c23)cccc4
	c1ccccc1-n(c2)nc(-c(cc3)ccc3C)c2/C=C4/C(=O)N(C(=O)S4)Cc5ccc([N+]([O-])=O)cc5
BindingDB_50138734_mol_1	CC(C)(C)c1ccc(cc1)C(=O)Nc2ccc(cc2)-n(c(=O)o3)nc3OC
BindingDB_51098581_3D	CCOc1nn(c(=O)o1)-c2cc(ccc2)OCc3ccccc3
BindingDB_50890312_3D	O=C(O)CC(\C=O)=C/C[C@@H]1C(=C)CC[C@H]([C@@]12C)C(C)(C)CCC2

BindingDB_51098600_3D	O=C(S1)NC(=O)\C1=C\c2c(-c3ccc(Cl)cc3)nn(c2)-c4ccccc4
BindingDB_51366108_3D	C1CCC(C)(C)[C@@H]([C@]12C)CCC(=C)[C@H]2C\C=C(\C=O)CC(=O)OCc3cn(nn3)Cc4c(cccc4)-c5ccccc5
BindingDB_50542126_3D	c1cc(O)c(OC)cc1/C=C(/C2=O)Cc(c23)ccc(c3)O
BindingDB_50337362_mol_1	COc1c(OC)cc(cc1OC)NC(=O)C(=O)c(c2)ccc3n(CC)c(c4c23)cccc4
BindingDB_51366109_3D	C[C@H](O)[C@H](O)c1cc(OC)c(O)cc1
BindingDB_50890314_3D	c1cc(O)c(OC)cc1/C=C(/C2=O)Cc(c23)ccc(c3)N
BindingDB_50890323_3D	c1ccccc1-n(c2)nc(-c3ccccc3)c2/C=C4/C(=O)N(C(=O)S4)Cc5ccc([N+]([O-])=O)cc5
BindingDB_50213683_mol_1	O=C(S1)NC(=O)\C1=C\c2c(-c3ccc(F)cc3)nn(c2)-c4ccccc4
BindingDB_50890322_3D	Oc1c(O)c(C(C)C)cc(c1[C@]234)[C@@H](OC2=O)C[C@H]3C(C)(C)CCC4
	O=C(S1)NC(=O)\C1=C\c2c(-c3ccc(cc3)OC)nn(c2)-c4ccccc4
Orlistat	CCCCCCC(C1=O)C(O1)CC(OC(=O)C(NC=O)CC(C)C)C/C=C\C/C=C\CCCCC
BindingDB_51366134_3D	CCCCCC[C@H](C1=O)[C@@H](O1)C[C@@H](OC(=O)[C@@H](NC=O)CC(C)C)CCCCCCCCCCC
BindingDB_50494715_3D	c1cc(O)c(OC)cc1/C=C(/C2=O)Cc(c23)ccc(c3)N(CCCCCCCCCCCCO)CCCCCCCCCCCCO
BindingDB_50494713_3D	CC(C)=CCC/C(C)=C\CC/C(C)=C\Cc1c(c(CC=C(C)C)c(O)cc1O)-c(c2)oc(c23)cc(O)cc3
BindingDB_50494714_3D	CC(C)=CCC/C(C)=C\Cc(c(O)cc1)c(c12)oc(c2)-c(cc3O)cc(c34)occ4
BindingDB_51366132_3D	CC(C)=CCC/C(C)=C\CC/C(C)=C\Cc1c(cc(O)cc1OC)-c(c2)oc(c23)cc(O)cc3
BindingDB_51366135_3D	C=CCCCCCCCCN(CCCCCCCCC=C)c(c1)ccc(c12)CC(/C2=O)=C\c3cc(OC)c(O)cc3
BindingDB_50303004_mol_1	c1cc(O)c(OC)cc1/C=C(/C2=O)Cc(c23)ccc(c3)NCCCCCCCCCCCCO
BindingDB_50494708_3D	CC(C)=CCC/C(C)=C\CC/C(C)=C\Cc1c(cc(O)cc1O)-c(c2)oc(c23)cc(O)cc3
BindingDB_51098586_3D	CC(C)=CCC/C(C)=C\Cc(c1O)c(O)cc(c12)O[C@@H](CC2=O)c(ccc3O)c(O)c3CC=C(C)C
BindingDB_	C1CCC(C)(C)[C@@H]([C@]12C)CCC(=C)[C@H]2C\C=C(\C=O)CC(=O)OCc3cn(nn3)Cc4ccc(F)cc4

50494712_3D	
BindingDB_51098582_3D	CC(C)=CCC[C@](C)(C=C1)Oc(c12)cc(cc2O)-c(c3)oc(c34)cc(O)cc4
BindingDB_51098577_3D	C1CCC(C)(C)[C@@H]([C@]12C)CCC(=C)[C@H]2C\C=C(\C=O)CC(=O)OCc3cn(nn3)Cc4ccc(Cl)cc4
BindingDB_51366130_3D	C1CCC(C)(C)[C@@H]([C@]12C)CCC(=C)[C@H]2C\C=C(\C=O)CC(=O)OCc3cn(nn3)Cc4ccc(cc4)SC
BindingDB_51098589_3D	c1cc(O)c(OC)cc1/C=C(/C2=O)Cc(c23)ccc(c3)N(CC[C@H](C)CCCC(C)C)CC[C@H](C)CCCC(C)C
BindingDB_50494707_3D	C1CCC(C)(C)[C@@H]([C@]12C)CCC(=C)[C@H]2C\C=C(\C=O)CC(=O)OCc3cn(nn3)Cc4ccc(cc4)OC
BindingDB_51098587_3D	CC(C)=CCC/C(C)=C\Cc(c(OC)cc(c1)O)c1/C=C/c2ccc(O)cc2
BindingDB_51098588_3D	C1CCC(C)(C)[C@@H]([C@]12C)CCC(=C)[C@H]2C\C=C(\C=O)CC(=O)OCc3cn(nn3)Cc4ccc([N+]([O-])=O)cc4
BindingDB_50494711_3D	C1CCC(C)(C)[C@@H]([C@]12C)CCC(=C)[C@H]2C\C=C(\C=O)CC(=O)OCc3cn(nn3)Cc(cc4)ccc4C(C)(C)C
BindingDB_51134709_3D	CC(C)=CCC/C(C)=C\Cc(c(O)cc1)c(c12)oc(c2)-c(c3CC=C(C)C)cc(O)cc3OC
BindingDB_51098599_3D	CCCCCC[C@@H](C1=O)[C@H](O1)C[C@@H](OC(=O)[C@H](NC=O)CC(C)C)CCCCCCCCCCC
BindingDB_51366127_3D	C1CCC(C)(C)[C@@H]([C@]12C)CCC(=C)[C@H]2C\C=C(\C=O)CC(=O)OCc3cn(nn3)Cc4c(Br)cc(F)cc4
BindingDB_51366128_3D	c1cc(O)c(OC)cc1/C=C(/C2=O)Cc(c23)ccc(c3)N(CC)CC
BindingDB_51409226_3D	C=CCCCCN(CCCCC=C)c(c1)ccc(c12)CC(/C2=O)=C\c3cc(OC)c(O)cc3
BindingDB_51366131_3D	CCCCCC[C@H](C1=O)[C@@H](O1)C[C@@H](OC(=O)c2ccc(O)cc2)CCCCCCCCCCC
BindingDB_51366123_3D	c1cc(O)c(OC)cc1/C=C(/C2=O)Cc(c23)ccc(c3)NCC[C@H](C)CCCC(C)C
Untitled_Molecule_000236	c1cc(O)c(OC)cc1/C=C(/C2=O)Cc(c23)ccc(c3)OCCC
BindingDB_50559541_3D	c1cc(Br)ccc1Cc(nc(c23)cccc3)n(c2=O)CC(=O)NNC(=O)c(c4)c(=O)oc(c45)cccc5
BindingDB_50559536_3D	CCCC(=O)OCC(OC(=O)CCC)COC(=O)CCC
BindingDB_	CCCC(=O)O[C@@H]1C[C@@H]([C@@H]12)C=CC2

429796_3D	
BindingDB_429813_3D	c1ccc(O)c(OC)c1CCc2cc(O)cc(c2)O
BindingDB_429814_3D	c1cc(O)ccc1CCC(=O)/C=C/C=C/c2ccc(O)cc2
BindingDB_429804_3D	c1cc(O)ccc1CCC(=O)/C=C/C=C/c2cc(OC)c(O)cc2
BindingDB_429794_3D	COc1cc(O)cc2ccc(c3c12)cc(O)c(c3)OC
BindingDB_429812_3D	c1c(O)cc(O)cc1CCc2cc(OC)c(O)cc2
Untitled	c1cc(O)ccc1\C=C\C(=O)/C=C/C=C/c2ccc(O)cc2
BindingDB_429799_3D	Oc1cc(OC)cc2CCc(c3c12)cc(O)cc3
BindingDB_50211956_mol_1	COc(c(c1)O)c(O)cc1CCc2ccccc2
BindingDB_429795_3D	c1cccc(O)c1CCc2cc(OC)cc(c2)O
BindingDB_429797_3D	c1cccc(O)c1CCc2cc(O)cc(c2)O
BindingDB_429810_3D	c1c(O)cc(OC)cc1CCc2cc(O)ccc2
442705	c1cc(O)ccc1\C=C\C(=O)OCCc2ccc(O)cc2
BindingDB_50381859_mol_1	COc1cc(O)cc2CCc(c3c12)cccc3O
BindingDB_429800_3D	c1cccc(c12)ccc(c2)[C@@H](C(=O)N)NC(=O)[C@H](c(c3)ccc(c34)cccc4)NC(=O)CCC(=O)Nc5ccc(cc5)OCCCC
BindingDB_429803_3D	c1cccc(O)c1CCc2cc(OC)cc(c2)OC
BindingDB_50381861_mol_1	c1cccc(O)c1CCc2cc(OC)c(OC)c(c2)OC
BindingDB_50381860_mol_1	c1ccccc1C[C@@H](C(=O)N)NC(=O)[C@H](Cc2ccccc2)NC(=O)CCC(=O)Nc3ccc(cc3)OCCCCCCC
BindingDB_50381858_mol_1	c1ccccc1C[C@@H](C(=O)N)NC(=O)[C@H](Cc2ccc(O)cc2)NC(=O)CCC(=O)Nc3ccc(cc3)OCCCC
BindingDB_51134702_3D	c1cc(O)ccc1C[C@@H](C(=O)N)NC(=O)[C@H](Cc2ccccc2)NC(=O)CCC(=O)Nc3ccc(cc3)OCCCC
BindingDB_51134706_3D	CCCCCC[C@H](C1=O)[C@@H](O1)C[C@H](OC(=O)[C@H](NC=O)CC(C)C)CCCCCCCCCCC
BindingDB_51134705_3D	CCCCCC[C@H](C1=O)[C@H](O1)C[C@@H](OC(=O)[C@@H](NC=O)CC(C)C)CCCCCCCCCCC
BindingDB_50274723_mol_1	CCCCCC[C@@H](C1=O)[C@H](O1)C[C@@H](OC(=O)[C@@H](NC=O)CC(C)C)CCCCCCCCCCC
BindingDB_51134701_3D	CCCCCC[C@H](C1=O)[C@@H](O1)C[C@@H](OC(=O)[C@H](NC=O)CC(C)C)CCCCCCCCCCC
BindingDB_51134710_3	CCCCCC[C@H](C1=O)[C@@H](O1)C[C@H](OC(=O)[C@@H](NC=O)CC(C)C)CCCCCCCCCCC

D	
BindingDB_51134704_3D	CCCCCC[C@@H](C1=O)[C@H](O1)C[C@H](OC(=O)[C@@H](NC=O)CC(C)C)CCCCCCCCCCC
BindingDB_50303006_mol_1	CCCCCC[C@@H](C1=O)[C@H](O1)C[C@H](OC(=O)[C@H](NC=O)CC(C)C)CCCCCCCCCCC
	CC(C)=CCC/C(C)=C/Cc(c(O)cc1)c(c12)oc(c2)-c(c3CC=C(C)C)cc(O)cc3O

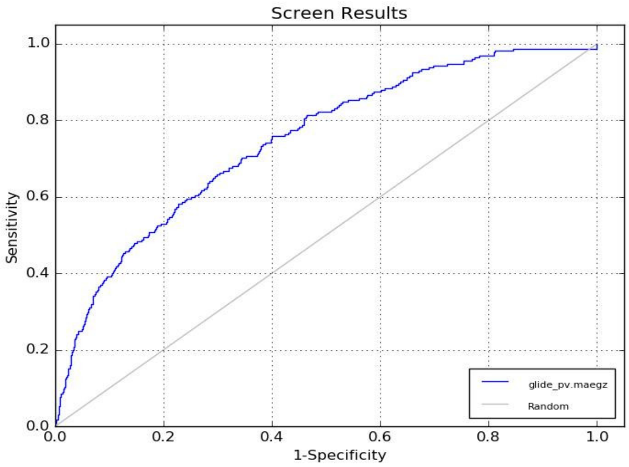

Figure S1. ROC Curve Comparing Glide XP Enrichment Performance to a Random Model. Receiver Operating Characteristic (ROC) curve for the enrichment study obtained using the Glide XP protocol (blue line) compared to a random model (gray line). The ROC curve demonstrates the trade-off between sensitivity (true positive rate) and Specificity (false positive rate).

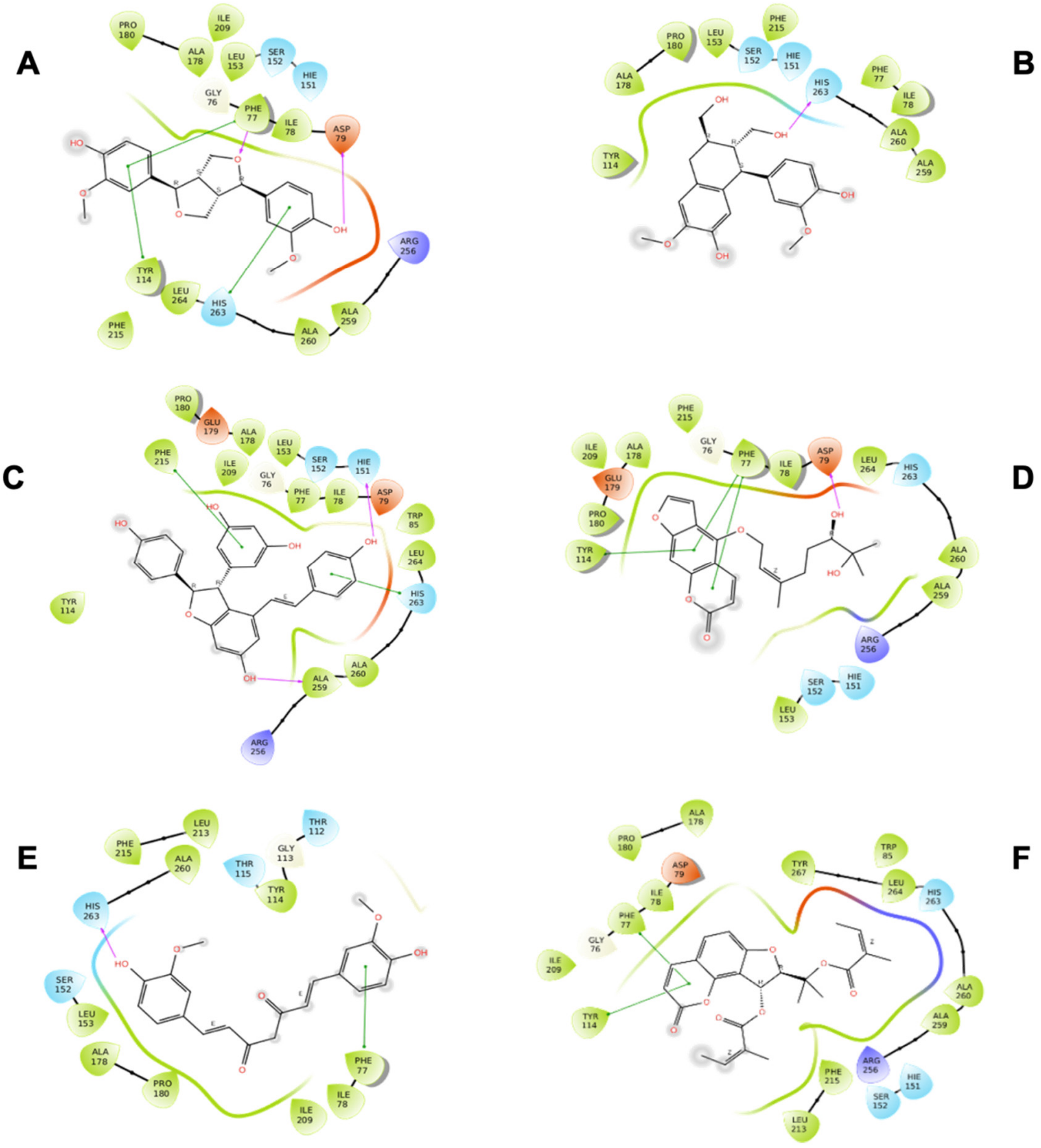

Figure S2: 2D Interaction Diagrams of Pancreatic Lipase with Selected Natural Inhibitors. 2D representations of PL complexed with **A)** Pinoresinol (PHUB001389), **B)** Isolariciresinol (PHUB001722), **C)** Ɛ-Viniferin (PHUB000318), **D)** Dihydroxybergamottin (PHUB000255), **E)** Curcumin (PHUB001408), and **F)** Archangelicin (PHUB000235). Hydrogen bonds and π-π stacking interactions are depicted as magenta and green lines, respectively. The figure was obtained by Maestro (Schrödinger Release 2023-1: Maestro, Schrödinger, LLC, New York, NY, 2023).

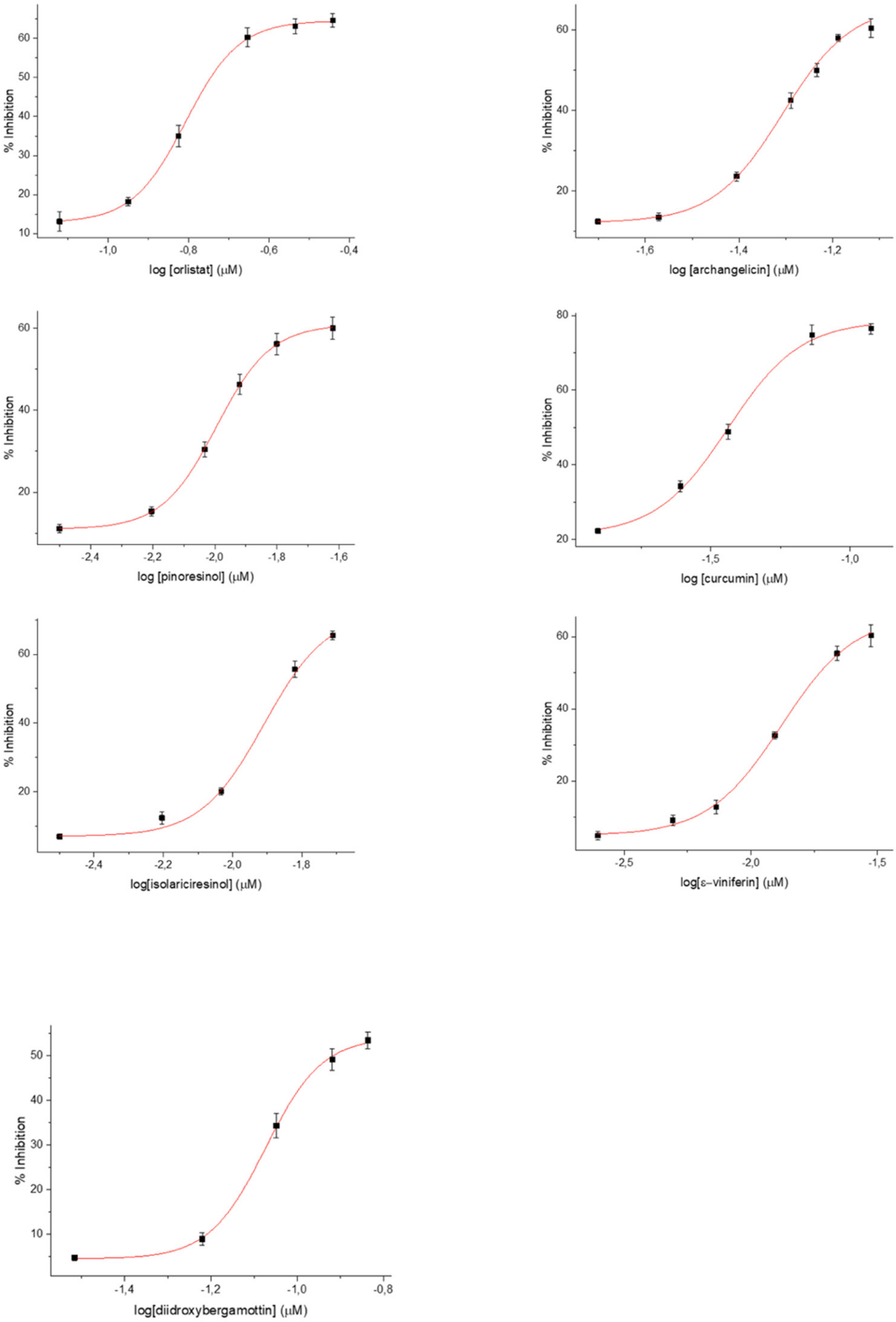

Figure S3: Dose–Response Curves of Orlistat and Selected Compounds for Pancreatic Lipase Inhibition. Dose-response curves for the pancreatic lipase inhibitory activity of orlistat and selected compounds. Data points are obtained according to equation (1) reported in the manuscript.

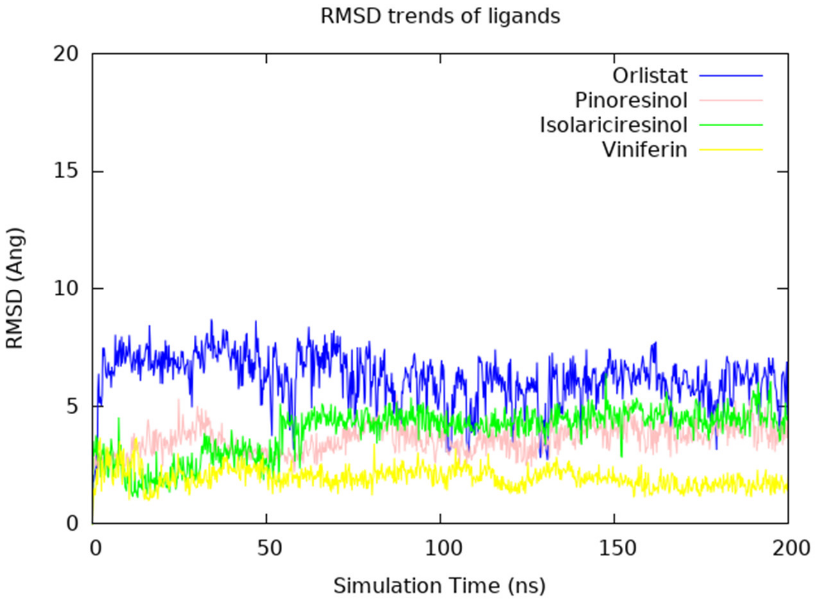

Figure S4. RMSD Trend of Selected Ligands in Complex with Pancreatic Lipase During MD Simulations. Root-mean-square deviation (RMSD) profiles of Orlistat (blue line), Pinoresinol (PHUB001389) (pink line), Isolariciresinol (PHUB001722) (green line), and Ɛ-Viniferin (PHUB000318) (yellow line) in complex with pancreatic lipase (PL). RMSD values were calculated for the ligand heavy atoms over the course of molecular dynamics (MD) simulations, following superposition of the protein backbone.
